# Exercise dose architecture as a precision-informed developmental framework in autism spectrum disorder: a narrative review of mechanisms, sex- and development-related heterogeneity, and clinical translation

**DOI:** 10.3389/fpsyt.2026.1865515

**Published:** 2026-07-15

**Authors:** Zhenqian Zhou, Dianhui Peng, Ping Peng, Xiaolin Li

**Affiliations:** 1 Hunan Agricultural University, Changsha, China; 2 Beijing Sport University, School of Art, Beijing, China; 3 Hebei Normal University, School of Physical Education, Shijiazhuang, China

**Keywords:** autism spectrum disorder, clinical translation, developmental stage, exercise dose, neuroplasticity, physical activity, precision intervention, sex-dependent heterogeneity

## Abstract

Autism spectrum disorder (ASD) is a heterogeneous neurodevelopmental condition characterized not only by differences in social communication and restricted or repetitive behaviors, but also by variable profiles of motor function, sleep, cognition, sensory processing, and adaptive participation. Exercise and structured physical activity have emerged as promising non-pharmacological interventions in ASD, with growing evidence supporting benefits across multiple outcome domains, including executive function, stereotyped behaviors, sleep, motor competence, social communication, and quality of life. However, this field is constrained by fragmented concepts, inconsistent dose documentation, lack of mechanistic integration, and inadequate clinical translation. Exercise is often treated as a generic intervention rather than as a well-defined and physiologically meaningful exposure. This narrative review proposes a precision-informed framework in which exercise in ASD is conceptualized as a structured developmental exposure whose potential therapeutic value may depend on dose architecture rather than modality labels alone. Beyond traditional descriptors such as frequency, intensity, time, and type, exercise dose in ASD should be understood as a multidimensional construct that also includes motor complexity, cognitive demand, social embeddedness, sensory load, predictability, progression rate, and adherence burden. We synthesize ASD-specific clinical evidence together with indirect evidence from broader exercise neuroscience and animal models, while explicitly distinguishing established clinical findings from candidate or hypothesis-generating mechanisms. We also examine how exercise responses may differ according to developmental stage, pubertal changes, gender-associated traits, and the lack of female representation in existing evidence. Overall, we investigate how efficacy findings can inform individualized exercise plans, mechanism-linked trials, and scalable interventions in real-world settings. Taken together, this review offers a conceptual and translational framework for future ASD exercise research, emphasizing standardized dose reporting, evidence-level distinctions, developmental stratification, safety monitoring, and clinically meaningful outcomes.

## Graphical Abstract

**Figure:**
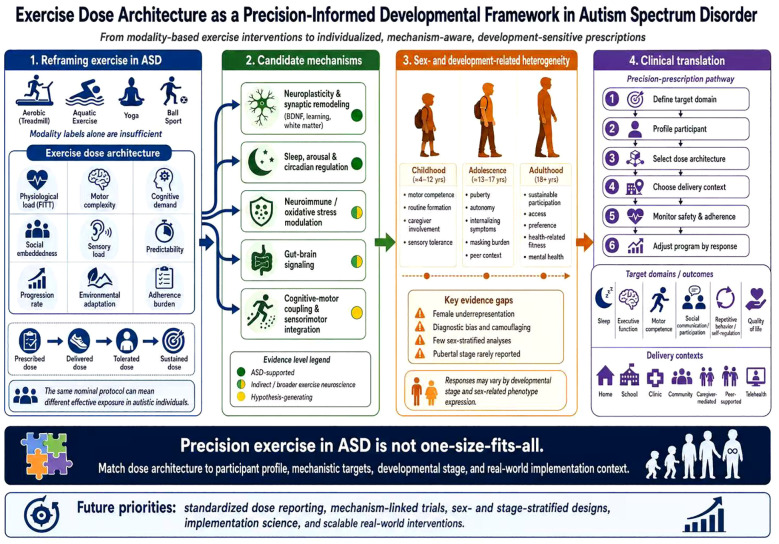
From modality-based exercise to precision dose architecture: an integrated framework for autism spectrum disorder.

## 1 Introduction

Autism spectrum disorder (ASD) is a heterogeneous neurodevelopmental condition characterized by differences in social communication, restricted or repetitive behaviors, and highly variable profiles of sensory processing, motor function, sleep, cognition, and adaptive participation (1–3). Although the clinical presentation of ASD is traditionally framed around core diagnostic features, it is increasingly clear that many of the functional burdens that shape daily life arise from broader interacting domains, including motor impairment, executive dysfunction, sleep disturbance, emotional dysregulation, low physical activity participation, and reduced opportunities for sustained social engagement (4–7). This expanded view has important implications for intervention science, because it suggests that effective non-pharmacological strategies may need to target not only core symptoms, but also the physiological and behavioral systems that constrain participation and long-term developmental adaptation (2, 4, 6).

In this context, exercise and organized physical activity are increasingly regarded as potentially important interventions for ASD. Over the past decade, systematic reviews and meta-analyses have shown that exercise-based interventions can improve a range of ASD-relevant outcomes, including motor performance, executive functions, stereotyped behaviors, sleep, and aspects of social functioning (4, 8–11). More recent integrative reviews further indicate that the benefits of exercise in autistic individuals are unlikely to be restricted to a single outcome domain, but instead may extend across behavioral, cognitive, physiological, and quality-of-life dimensions (2, 12). At the same time, the literature also shows considerable heterogeneity in outcomes, intervention formats, and participant characteristics, making it increasingly difficult to sustain the simplistic question of whether exercise “works” for ASD in a general sense (4, 8, 10, 12).

A major limitation of the existing literature is that exercise is often treated as a generic intervention rather than as a structured and measurable exposure. In many studies, programs are primarily compared according to modality, such as aerobic exercise, aquatic exercise, ball games, or mind-body activities, without adequately specifying which elements of the intervention are most therapeutically important (2, 13). Moreover, in ASD, the effective ‘dose’ of exercise extends beyond frequency, intensity, duration, and type alone, potentially involving motor complexity, social integration, sensory demands, predictability, progression rate, and implementation burden (11). This issue is particularly important because different intervention architectures may engage distinct mechanistic pathways, including neuroplasticity, sleep-arousal regulation, neuroimmune modulation, gut-brain interactions, and cognitive-motor coupling (14–16). Focusing on dose may offer more explanatory power than focusing on modality.

Another important issue is the limited integration of developmental stage and sex- or gender-related differences in ASD exercise evidence. Although the ASD literature as a whole remains male-skewed, emerging work suggests that early phenotypic sex differences may be smaller than previously assumed, while later divergence may arise through developmental trajectories, pubertal transition, social masking, diagnostic bias, and differences in co-occurring internalizing burden (17–20). Likewise, evidence on exercise in ASD has mainly focused on children, with substantially less evidence available for adolescents, young adults, and especially autistic females (6, 17). Thus, the field currently lacks sufficient evidence to determine whether exercise responses differ according to gender-related phenotype expression, developmental timing, or pubertal status. This limitation is crucial: a program effective for a prepubescent boy may pose different social, sensory, and emotional challenges for a teenage girl or an autistic young adult (18–21).

Clinical translation remains underdeveloped. While efficacy data have accumulated, many studies still rely on short intervention periods, modest sample sizes, limited mechanistic characterization, and outcome measures centered on broad symptom change instead of on clinically meaningful functional targets (2, 12). At the same time, real-world translation is restricted by participation barriers, family burden, limited access to tailored programs, inconsistent use of behavior-change approaches, and the absence of trial designs centered on implementation (7, 22–24). These issues suggest that the future direction of this field should prioritize a more detailed framework that connects exercise dose architecture, mechanistic targets, participant characteristics, and the context of implementation, rather than just broad claims of efficacy.

Thus, this review does not claim that exercise is already an established precision intervention for ASD; rather, it proposes that exercise should be studied and reported as a precision-informed developmental exposure whose effects may vary according to dose structure, participant characteristics, developmental stage, and implementation context. Specifically, this review aims to: (1) redefine exercise dose architecture as a multidimensional model relevant to ASD; (2) outline candidate mechanistic pathways to explain how exercise could affect the biology and behavior associated with ASD; (3) investigate how exercise responses differ by sex and developmental stage; (4) evaluate the shift from efficacy studies to personalized exercise prescriptions in clinical environments; and (5) propose future directions for precision exercise science. By shifting the focus from broad efficacy claims to dose specification, evidence directness, subgroup heterogeneity, and clinical feasibility, this review aims to provide a more transparent framework for hypothesis generation, trial design, and cautious clinical translation.

## 2 Methods

### 2.1 Review design and scope

This review was designed as a narrative review with a proposed conceptual framework, rather than as a systematic review, scoping review, or meta-analysis. The objective was not to estimate pooled intervention effects, but to integrate heterogeneous evidence on exercise dose, ASD-related outcomes, candidate mechanisms, developmental heterogeneity, sex-related evidence gaps, and clinical translation. The narrative format was chosen because the reviewed literature includes randomized trials, systematic reviews, biomarker studies, neuroimaging studies, animal-model experiments, developmental studies, implementation studies, and clinical feasibility reports that cannot be meaningfully combined within a single pooled-effect model. To improve transparency, the present revision follows key principles of structured narrative reporting, including clear review questions, explicit search sources, inclusion and exclusion criteria, evidence interpretation rules, and a simplified study selection flow. Because of substantial heterogeneity in exercise modalities, participant characteristics, outcome measures, developmental stages, and mechanistic endpoints, this review adopt a narrative review approach to integrate evidence from clinical trials, systematic reviews, mechanistic studies, neurodevelopmental literature, and clinical translation perspectives into a unified framework.

This review addresses five primary questions: (1) In ASD, how does exercise dose architecture transcend traditional FITT (Frequency, Intensity, Time, and Type) descriptions (2) Which biological and behavioral mechanisms may mediate exercise-related effects; (3) How do gender-related phenotypic expressions and developmental stages influence exercise responses; (4) How can exercise interventions be translated into clinically meaningful and implementable individualized exercise prescriptions; (5) What methodological issues should be prioritized in next-generation precision exercise research for ASD. A systematic review or meta-analysis was not selected because the primary aim was conceptual integration rather than quantitative effect estimation. Existing ASD exercise studies differ substantially in participant age, diagnostic characterization, support needs, exercise modality, intervention duration, dose reporting, comparator condition, outcome measures, and mechanistic endpoints. A scoping review was also not selected because the aim was not only to map the breadth of available literature, but to propose and refine a dose-architecture framework for future mechanistic and translational research. Therefore, the manuscript should be interpreted as a structured narrative review with a hypothesis-generating framework, not as a definitive evidence-grading document for clinical guidelines.

### 2.2 Literature search strategy

Targeted literature searches were conducted in PubMed, Web of Science, Scopus, PsycINFO, and Google Scholar from database inception to April 2026. The search was not prospectively registered because the manuscript was designed as a narrative review; however, the search strategy was reconstructed and reported in greater detail to improve transparency and reproducibility. Key search terms included the following keywords and their combinations: “autism spectrum disorder”, “ASD”, “autistic individuals”, “exercise”, “physical activity”, “exercise intervention”, “exercise dose”, “exercise prescription”, “motor skill”, “executive function”, “sleep”, “social communication”, “repetitive behavior”, “quality of life”, “neuroplasticity”, “ BDNF”, “white matter”, “neuroinflammation”, “oxidative stress”, “gut microbiota”, “gut-brain axis”, “sex differences”, “female autism”, “camouflaging”, “puberty”, “developmental stage”, “adherence”, “telehealth”, and “implementation science”. An example PubMed search string was: (“autism spectrum disorder” OR ASD OR autistic) AND (exercise OR “physical activity” OR “exercise intervention” OR “exercise prescription” OR “motor skill training” OR aquatic OR basketball OR yoga OR exergaming) AND (dose OR dosage OR intensity OR frequency OR duration OR FITT OR adherence OR feasibility OR implementation OR sleep OR “executive function” OR stereotyped OR “social communication” OR “quality of life”). Additional searches combined ASD terms with mechanistic keywords, including BDNF, neuroplasticity, white matter, neuroinflammation, oxidative stress, gut microbiota, gut-brain axis, melatonin, actigraphy, puberty, sex differences, female autism, and camouflaging. Google Scholar was used primarily for citation tracking and identification of recent or cross-disciplinary literature, rather than as the main source for exhaustive record counting. In addition, this study supplemented the literature by tracing references and conducting citation tracking of relevant reviews, meta-analyses, randomized controlled trials, mechanistic studies, and clinical intervention studies. The literature screening prioritized recent systematic reviews and meta-analyses, ASD-specific exercise intervention studies, and studies that could provide mechanistic or translational insights regarding exercise dose, developmental heterogeneity, or clinical prescription.

### 2.3 Inclusion and exclusion criteria

Eligible sources included the following types of literature: (1) Systematic reviews and meta-analyses examining the effects of exercise or physical activity interventions in ASD; (2) Randomized controlled trials and controlled intervention studies involving exercise, physical activity, motor skills training, aquatic exercise, sports-based interventions, or structured exercise programs; (3) Studies reporting ASD-related outcomes, including executive function, motor skills, sleep, stereotyped or repetitive behaviors, social communication, adaptive functioning, quality of life, or participation in physical activity; (4) Mechanistic studies involving neuroplasticity, BDNF, neuroimaging, inflammatory markers, sleep-related physiology, gut microbiota, or cognitive-motor coupling; (5) Studies discussing gender differences in ASD, autism in females, camouflaging/masking behaviors, adolescence, developmental stages, or age-related heterogeneity; (6) Studies involving adherence, family feasibility, telemedicine, caregiver-mediated interventions, and barriers to the implementation of exercise prescriptions.

Exclusion criteria include: studies not directly related to ASD; studies not involving exercise or structured physical activity; studies discussing general physical activity participation without clinical or developmental significance; and studies unable to provide sufficient information for conceptual synthesis. Animal studies were included only when they involved ASD-like phenotypes or exercise-responsive biological pathways, with their limitations regarding clinical translation fully considered in the interpretation.

To reduce selection bias, evidence inclusion was guided by predefined conceptual domains rather than by whether studies reported positive exercise effects. Studies reporting null, inconsistent, or adverse findings were retained when they were relevant to ASD exercise response, dose interpretation, feasibility, safety, or mechanistic plausibility. Recent systematic reviews and meta-analyses were used to anchor clinical efficacy claims, whereas individual studies were used mainly to illustrate dose structure, mechanistic readouts, subgroup heterogeneity, or translational barriers. When multiple studies addressed the same outcome domain, priority was given to ASD-specific systematic reviews, meta-analyses, randomized controlled trials, controlled intervention studies, and studies with clearly described exercise dose and participant characteristics.

### 2.4 Evidence appraisal and risk-of-bias considerations

No formal risk-of-bias assessment tool was applied because this manuscript was not designed as a systematic review and included heterogeneous evidence types that were not suitable for a single appraisal instrument. Instead, we applied an evidence-directness approach. ASD-specific randomized controlled trials and ASD-focused meta-analyses were treated as the strongest sources for clinical efficacy. ASD-specific biomarker, neuroimaging, sleep, and physiological studies were treated as supportive but not definitive mechanistic evidence. Animal-model studies and non-ASD exercise neuroscience were used only to support biological plausibility and were not interpreted as direct evidence of clinical efficacy in autistic individuals. Throughout the revised manuscript, mechanistic statements were therefore labeled as established, ASD-supported, indirect, or hypothesis-generating according to the directness of the evidence.

### 2.5 Narrative synthesis methodology

This review uses a concept-driven synthesis approach to integrate the literature. The evidence is organized around three levels. First, clinical and behavioral evidence is used to assess which ASD-related outcomes may respond to exercise interventions. Second, mechanistic evidence is used to identify candidate pathways through which different exercise dose structures may influence neurodevelopmental and behavioral outcomes. Third, developmental and translational evidence is used to analyze how gender-related phenotypic expression, adolescence, age, sensory tolerance, family environment, and implementation barriers affect the feasibility of movement and response to intervention.

This review does not treat exercise modality as the sole or primary unit of analysis, but rather emphasizes the “exercise dose architecture”. This framework encompasses not only physiological parameters such as intensity, frequency, duration, and type, but also exercise complexity, cognitive demands, degree of social embedding, sensory load, predictability, progression rate, environmental adaptation, and adherence burden. This framework is used to explain why seemingly similar exercise interventions may yield different outcomes, and why the same nominal protocol may constitute different effective doses across individuals. **Table 1** summarizes key ASD exercise studies and reviews included in the narrative synthesis.

**Table 1:** Summary of key ASD exercise studies and reviews included in the narrative synthesis.

Study	Design/evidence type	Participants and female representation	Exercise modality/intervention focus	Dose/duration	Main outcomes	Major findings	Relevance to this review/limitations	Ref.
Rivera et al., 2025	Integrative review of clinical trials	evidence mainly from children and adolescents; adults and females underrepresented	Exercise interventions across clinical trials	Heterogeneous across studies	Social communication, executive function, sleep, physical health, broader functioning	Exercise programs appear beneficial for several functional domains, especially in children and adolescents	Supports broad clinical relevance of exercise, but highlights heterogeneity and limited evidence for adults, girls/women, and high-support-needs subgroups	(5)
Healy et al., 2018	Meta-analysis	Youth with ASD; sex distribution not consistently summarized across included studies	Physical activity interventions	Heterogeneous modalities and durations	Motor skills, social functioning, stereotyped behavior, behavioral outcomes	Physical activity interventions showed generally positive effects in youth with ASD	Foundational evidence for efficacy; limited by heterogeneity and incomplete dose reporting	(3)
Shahane et al., 2024	Systematic review	Young adults with ASD, 19–30 years; 22 studies, 763 participants; female representation not consistently emphasized	Physical activity and exercise-based interventions	Heterogeneous	Physical fitness, motor skills, psychological function, quality of life, physical activity engagement, core symptoms	Strongest evidence for physical fitness, followed by motor skills, psychological function, and quality of life; insufficient evidence for physical activity engagement or core autism symptoms	Supports lifespan argument; shows adult evidence remains sparse and less mechanistically developed	(6)
Liang et al., 2022	Systematic review and meta-analysis	Children and adolescents with ASD; female proportion not consistently reported	Acute and chronic exercise interventions	Varied; chronic exercise analyzed separately	Executive functions: inhibition, cognitive flexibility, working memory	Chronic exercise had small-to-moderate positive effects on overall executive function, cognitive flexibility, and inhibitory control; working memory effect was non-significant	Supports domain-specific prescription for executive function; also shows outcome-specific inconsistency	(7)
Liang et al., 2024	Systematic review and meta-analysis	Children and adolescents with ASD; sex distribution not consistently reported	Physical activity interventions targeting sleep	Heterogeneous	Sleep quality and sleep-related outcomes	Physical activity interventions were associated with improvements in sleep among children and adolescents with ASD	Supports sleep/arousal pathway; limited by variation in sleep measures and intervention protocols	(20)
Wu et al., 2024	Systematic review and meta-analysis	Individuals with ASD; sex and developmental distributions varied across included studies	Physical exercise therapy	Heterogeneous	ASD-related symptoms and functional outcomes	Physical exercise therapy showed evidence of benefit, but effects varied across outcomes and intervention types	Supports general efficacy claims, but reinforces need for dose-specific interpretation	(10)
Yang and Li, 2025	Systematic review and RCT meta-analysis	Individuals with ASD; sex distribution not consistently summarized	Physical activity interventions and components	Heterogeneous	Repetitive stereotyped behaviors	Physical activity interventions may reduce repetitive stereotyped behaviors; intervention components appear relevant	Supports symptom-domain-specific analysis; limited by heterogeneity in intervention components	(8)
Ataíde et al., 2026	Systematic review of exercise prescription	Individuals with ASD; sex representation varied; not consistently synthesized	Exercise prescription across ASD studies	Heterogeneous	Exercise type, prescription parameters, quality of life and health-related outcomes	Evidence supports exercise benefits, but prescription guidance remains limited	Directly supports the need for standardized exercise prescription and dose reporting	(9)
Tu et al., 2025	Mechanistic review	Autistic individuals; includes ASD-specific and broader mechanistic evidence	Exercise-related neurobiological mechanisms	Not applicable	Neuroplasticity, BDNF, neuroimmune modulation, brain networks	Exercise may influence ASD-related outcomes through multiple neurobiological pathways	Supports mechanism section; mechanisms should be treated as candidate pathways rather than established causal chains	(12)
Cai et al., 2020	Controlled intervention study with neuroimaging	29 children with ASD aged 3–6 years; control group 13 boys/1 girl, exercise group 12 boys/3 girls	Mini-basketball training program	12 weeks; 5 sessions/week; 40 min/session	Social communication, white matter integrity	Improved social communication and altered white matter integrity in several tracts	Strong ASD-specific human evidence linking exercise with behavioral and neuroimaging outcomes; small sample and male-skewed composition	(35)
Wang et al., 2020	Controlled intervention study	33 preschool children with ASD; control group 13 boys/2 girl, exercise group 15 boys/3 girls	Mini-basketball training program	12 weeks; 5 sessions/week; 40 min/session	Executive function, core symptoms	Mini-basketball training improved executive function and core symptoms in preschool children with ASD	Supports cognitive-motor coupling and socially embedded motor complexity; limited by preschool sample and modality-specific design	(34)
Ludyga et al., 2024	Randomized cross-over acute exercise study	29 children with ASD; final sample all boys	Single bout of moderate aerobic cycling	20 min acute exercise	Affective response inhibition, gaze fixation, socio-emotional processing	A single moderate exercise bout did not improve affective response inhibition and temporarily worsened ASD-specific socio-emotional processing	Essential null/adverse-response evidence; supports distinction between acute and chronic exercise effects	(23)
Zhao et al., 2024	Randomized controlled parallel study	30 children with ASD; final sample all boys	Aquatic exercise	12 weeks	Executive function, BDNF	Improved inhibitory control, cognitive flexibility, and BDNF levels; working memory did not significantly improve	Supports neuroplasticity-related mechanism; all-male sample limits sex-related generalizability	(33)
Ge et al., 2026	Intervention study with eye-tracking/brain-related outcomes	30 children with ASD aged 4–12 years; control group 12 boys/2 girl, exercise group 15 boys/1 girls	Group basketball-based motor skill learning	12 weeks; 5 sessions/week; 40 min/session	Joint attention, white matter changes	Group motor skill learning improved joint attention and was associated with brain changes	Supports social-motor and cognitive-motor coupling; requires cautious causal interpretation	(48)
Tse et al., 2022	Randomized exercise intervention	55 children with ASD, aged 8–12 years	Morning jogging	12 weeks; 30 min/session	Actigraphy-assessed sleep, sleep log, melatonin, behavioral functioning, repetitive behavior	Improved sleep efficiency, wake after sleep onset, melatonin level, and behavioral functioning; effects were not clearly sustained at follow-up	Strong ASD-specific evidence for sleep/arousal and melatonin-related pathway; follow-up durability limited	(49)
Ansari et al., 2021	Randomized controlled trial	40 boys with ASD, aged 6–14 years; 0% female	Water-based exercise	10 weeks; 2 sessions/week; 60 min/session	Sleep habits, IL-1β, TNF-α	Improved sleep quality and reduced serum IL-1β and TNF-α	Supports sleep and neuroimmune candidate pathway; all-male sample and peripheral biomarkers limit generalization	(51)
Tse et al., 2024	Randomized comparative intervention study	62 children with ASD; 50 boys/12 girls	Cycling, melatonin, combined intervention, placebo	2 weeks; 5 sessions/week; 60 min/session	Sleep efficiency, sleep onset latency, sleep duration, wake after sleep onset	Physical exercise and melatonin showed similar effectiveness in improving sleep quality; no significant differences among active interventions	Supports integration of exercise into sleep-focused care; does not establish exercise superiority over melatonin	(50)
Güeita-Rodríguez et al., 2021	Mixed-methods study	6 children with ASD; 5 boys/1 girls	Aquatic therapy	28 weeks; 2 sessions/week; 60 min/session	Social competence, quality of life	Aquatic therapy was associated with improvements in social competence and quality of life	Supports sensory-adapted and quality-of-life-oriented intervention design	(79)
García-Villamisar 2010	Intervention study	Individuals with ASD	Leisure program	48 weeks	Quality of life, stress	Leisure-based intervention improved quality of life and stress-related outcomes	Supports broader participation and well-being outcomes beyond core symptom scores	(80)
Cai et al., 2025	Systematic review and meta-analysis	Individuals with ASD; sex distribution varied across included studies	Exercise intervention	Heterogeneous	Cognitive function, quality of life	Exercise interventions were associated with improvements in cognitive function and quality of life	Supports clinically meaningful functional endpoints; limited by heterogeneity of included interventions	(74)
Columna et al., 2021	Randomized feasibility trial	15 families of children with ASD aged 4–11 years	Parent-mediated fundamental motor skill intervention	10 weeks; workshop or home-based format	Fundamental motor skills, feasibility, retention, safety, acceptability	Parents could facilitate children’s FMS development	Supports family-mediated implementation and safety reporting; small feasibility sample	(77)
Prieto et al., 2023	Randomized parent-mediated physical activity intervention	31 parent-child dyads; autistic children aged 4–11 years; 16 boys/9 girls	Parent-mediated physical activity; workshop, online, or control	12 weeks;	Fundamental motor skills, parent-mediated delivery	Parent-mediated physical activity showed promise for improving children’s fundamental motor skills	Supports scalable caregiver-mediated prescription; feasibility and generalizability remain limited	(82)
Shen et al., 2024	Randomized controlled trial	34 children with ASD; mean age 15.7 years; control group 9 boys/8 girl, exercise group 10 boys/7 girls	Telehealth-based parental support to increase MVPA	24 weeks; 3 sessions/week; 30 min/session	Moderate-to-vigorous physical activity, sleep quality	Telehealth plus parental support improved physical activity and sleep quality	Supports remote/hybrid implementation; sample size modest	(91)
Lee et al., 2022	Feasibility randomized controlled trial	Adults with ASD; 15 female/9 male	Gamified mobile app using behavior-change techniques	8 weeks	Physical activity, sedentary time, anxiety, feasibility	Gamified mHealth approaches may support physical activity but require careful attention to anxiety, routine disruption, and acceptability	Supports adult implementation science and digital delivery; feasibility-stage evidence only	(78)
Okkenhaug et al., 2024	Scoping review	Children and youth with ASD; female representation not consistently pooled	Barriers and facilitators for physical activity	Not applicable	Participation barriers, facilitators, environmental and family factors	Physical activity participation is shaped by individual, family, social, and environmental barriers	Supports adherence burden and environmental adaptation dimensions	(18)
Parsons et al., 2025	Systematic review	Autistic individuals; sex distribution varied across included studies	Behavior-change techniques in physical activity interventions	Not applicable	Physical activity behavior, intervention components	Behavior-change techniques are inconsistently integrated into ASD physical activity interventions	Supports implementation and adherence framework; not a direct efficacy trial	(26)

Female representation should be reported as exact numbers and percentages wherever available. For systematic reviews and meta-analyses, use “not consistently reported” only when the review does not provide a pooled sex distribution.

ASD, autism spectrum disorder.

### 2.6 Principles of evidence interpretation

Given the substantial heterogeneity in existing evidence regarding study design, sample characteristics, and outcome measures, this paper interprets conclusions based on the strength and directness of the evidence. ASD-specific randomized controlled trials and meta-analyses are considered the strongest sources of evidence for assessing clinical efficacy. Biomarker, neuroimaging, and physiological studies are used to support the plausibility of mechanisms when available. Animal studies and broader evidence from exercise neuroscience are used to cautiously support biological hypotheses but are not considered direct evidence of clinical efficacy for ASD. Therefore, unless ASD-specific intervention studies provide direct support, this paper defines most mechanistic statements as candidate pathways rather than established causal mechanisms.

## 3 Findings

### 3.1 Reframing exercise in ASD: from broad intervention to tailored dosage

#### 3.1.1 Why this field needs to move beyond the question of whether exercise benefits ASD

In the last ten years, the question of whether exercise benefits individuals with ASD has become insufficient as a research framework. Multiple systematic reviews and meta-analyses now indicate that structured physical activity and exercise interventions can improve several ASD-relevant domains, including motor performance, stereotyped or repetitive behaviors, social functioning, executive functions, sleep, and selected psychological outcomes (4, 8–10, 25, 26). More recent syntheses have reinforced this overall direction of effect, showing benefits for core symptoms or associated functional domains in children and adolescents, while also confirming that effects are not uniform across all outcomes or populations (2, 10, 11, 26). In summary, the literature suggests that exercise should not be viewed only through the lens of efficacy, but rather as a complex issue involving varied responses.

The unresolved question is not if exercise is beneficial, but rather which exercise dose is beneficial, for which individuals, under what developmental and behavioral circumstances, and through which potential mechanisms. This change is essential as current evidence suggests that the influence of exercise differs according to symptom domain, age group, duration of intervention, and program structure. For example, in young adults with ASD, the strongest evidence currently supports gains in physical fitness, motor skills, psychological function, and quality of life, whereas evidence for improving habitual physical activity levels and core autism symptoms is much less consistent (6). Likewise, acute and chronic exercise cannot be assumed to act in the same manner: although chronic interventions often show favorable effects, a randomized cross-over study found that a single 20-minute bout of moderate aerobic exercise did not improve affective response inhibition and transiently worsened aspects of socio-emotional processing in children with ASD (23). These findings suggest that the field should move beyond broad efficacy claims to a more detailed model of exercise response.

Another reason to move beyond the “does it work”? question is that seemingly positive results may obscure fundamentally different therapeutic processes. Improvements in executive function, sleep, repetitive behaviors, and social communication should not be regarded as equivalent indicators of a single intervention’s impact (8, 9, 25, 28). Rather, they may reflect partly distinct neurobehavioral pathways that are activated differently by different categories of exercise. Once this is acknowledged, exercise in ASD is viewed not merely as a typical supplementary therapy, but also as a tailored neurobehavioral intervention whose impact depends on its dosage and administration.

#### 3.1.2 Exercise dose architecture is a multifaceted concept in ASD

In the context of ASD, exercise dose should be reported not only through conventional FITT variables—frequency, intensity, time, and type—but also through ASD-relevant task, sensory, social, and implementation dimensions that shape the effective exposure received by the participant (12, 13). While those dimensions are still important, they do not completely represent the real exposure experienced by autistic participants. ASD-relevant exercise dose is more appropriately viewed as a multidimensional construct that includes not only physiological load, but also motor complexity, cognitive demand, social embeddedness, sensory load, predictability, environmental adaptation, progression rate, and adherence burden (12, 13, 29). A 30-minute session of moderate exercise, for example, may represent very different intervention doses depending on whether it consists of repetitive treadmill walking, a rule-based team ball game, aquatic activity with altered sensory input, or a highly scaffolded mind-body program with low social uncertainty.

This broader view is increasingly supported by ASD-specific evidence. Subgroup analyses from meta-analytic work suggest that exercise effects on disordered social communication are shaped by program composition, frequency, and duration, with multi-component programs, moderate-to-high frequency, and longer intervention periods showing stronger effects in some syntheses (24). Meta-analyses on repetitive stereotyped behaviors have stressed that both exercise participation and the specifics of the intervention are critical for clinical findings (8). Although these findings do not suggest a single ‘optimal dose’, they highlight that exercise dose architecture in ASD is complex, involving a combination of biological, behavioral, and contextual elements.

Critically, dose in ASD should be interpreted in relation to individual traits and participation context. Systematic reviews of physical activity correlates and barriers show that age, family support, environmental opportunities, social demands, and ASD-related participation barriers all shape whether a nominally prescribed exercise program becomes a tolerable, repeatable, and therapeutically meaningful dose (7, 22, 23). In other words, the administered dose differs from what the protocol specifies. For ASD, dose should be considered as a combination of delivered exposure, participant suitability, and ongoing engagement, rather than merely session duration or intensity.

For future studies, we define exercise dose architecture as the combination of physiological load, motor complexity, cognitive demand, social embeddedness, sensory load, predictability, progression rate, environmental adaptation, and adherence burden. These dimensions should be treated as reportable and, where possible, quantifiable intervention features rather than descriptive afterthoughts (12, 13, 22, 23, 30, 31).

Physiological load may be indexed by heart rate, percentage of heart-rate reserve, rating of perceived exertion, accelerometry, or metabolic-equivalent estimates (13, 30–33). Motor complexity may be indexed by balance requirements, bilateral coordination, object-control demands, task sequencing, and motor-skill assessments (5, 13, 34, 35). Cognitive demand may be indexed by rule switching, inhibition requirements, dual-task load, decision frequency, and working-memory demands (8, 36, 37). Social embeddedness may be coded as individual, dyadic, small-group, cooperative, competitive, or team-based activity (13, 15, 22, 23, 38, 39). Sensory load may be characterized by auditory noise, visual complexity, tactile input, vestibular demand, crowding, lighting, and transition demands (13, 22, 40, 41). Predictability and progression may be documented through session structure, visual schedules, novelty, progression rules, and weekly increases in intensity or task difficulty (13, 23, 42, 43). Adherence burden should be documented through attendance, dropout, caregiver burden, participant distress, and adverse-event monitoring (22, 23, 42–44). **Table 2** provides an operational framework for reporting and quantifying exercise dose architecture in ASD, including physiological, motor, cognitive, social, sensory, progression-related, environmental, adherence-related, and safety-related dimensions.

**Table 2:** Operational framework for exercise dose architecture in ASD.

Dose-architecture dimension	Operational definition	Possible indicators or proxies	Suggested measurement/coding approach	Relevance for ASD exercise prescription	Literature support
Physiological load	Cardiometabolic and neuromuscular intensity imposed by the activity	Heart rate, percentage of heart-rate reserve, rating of perceived exertion, accelerometry-derived activity intensity, metabolic-equivalent estimates	Wearable heart-rate monitoring; HRR calculation; Borg RPE scale; accelerometry; MET-based activity classification	Determines arousal, fatigue, cardiovascular load, sleep-related effects, and fitness adaptation; should be adjusted for tolerance and baseline fitness	(13, 38, 45, 46)
Frequency	Number of exercise sessions delivered within a defined period	Sessions per week; weekly accumulated exercise exposure	Protocol log; attendance sheet; caregiver or instructor record	Higher frequency may support routine formation and repeated biological exposure, but excessive frequency may increase family burden or refusal	(12, 13, 28)
Session duration	Length of each exercise exposure	Minutes per session; active exercise time; rest-adjusted active time	Instructor log; wearable activity recording; direct observation	Duration affects total dose and tolerability; active time should be distinguished from total session time	(12, 13, 55)
Intervention duration	Total length of the program	Number of weeks; total sessions; total accumulated exposure	Trial protocol; intervention log	Chronic exposure is more relevant for neuroplasticity, sleep, motor learning, and behavioral adaptation than isolated acute bouts	(8, 9, 12, 14, 27)
Exercise type/modality	Nominal form of exercise or physical activity	Aerobic exercise, aquatic exercise, ball games, martial arts, yoga, exergaming, motor-skill training, family-based physical activity	Modality classification with detailed intervention description	Useful for basic classification, but insufficient alone because the same modality may impose different motor, social, sensory, and cognitive demands	(12, 13, 54, 55)
Motor complexity	Degree of coordination, motor planning, balance, sequencing, and object-control demand	Balance requirements; bilateral coordination; locomotor skills; object-control skills; postural control; task sequencing	TGMD, BOT-2, MABC-2, direct task analysis, instructor-rated motor complexity	Relevant for motor competence, sensorimotor integration, imitation, action planning, and cognitive-motor coupling	(5, 13, 15, 37, 47, 48, 50, 58)
Cognitive demand	Executive-control load required during exercise	Rule switching; inhibition; working memory; decision frequency; dual-task demands; response selection	Task analysis; rule-change coding; dual-task coding; executive-function outcomes such as inhibition and cognitive flexibility	Especially relevant when targeting executive function, attention control, behavioral regulation, or flexible responding	(7, 33, 34, 36, 43, 73)
Social embeddedness	Degree and type of interpersonal interaction required during the activity	Individual, dyadic, small-group, cooperative, competitive, team-based, peer-assisted, caregiver-mediated	Social-demand coding; group-structure coding; observation of shared attention, turn-taking, cooperation, and peer interaction	May support social communication and participation, but may also increase social stress, masking burden, or refusal in some participants	(11, 18, 26, 35–37, 48, 60)
Sensory load	Auditory, visual, tactile, vestibular, proprioceptive, and environmental sensory demands imposed by the activity	Noise, lighting, crowding, water pressure, tactile contact, visual complexity, vestibular stimulation, transition demands	Sensory environment checklist; caregiver report; participant tolerance rating; observation of sensory distress	Central for feasibility and adherence in ASD; sensory overload may convert a physiologically appropriate dose into an intolerable intervention	(11, 18, 38, 39, 79)
Predictability	Degree to which the session structure, transitions, rules, and environment are stable and foreseeable	Fixed routine, visual schedule, consistent instructor, repeated sequence, clear transitions, novelty level	Session-structure coding; visual-schedule documentation; transition log; participant distress/refusal log	High predictability may reduce anxiety and behavioral dysregulation; controlled novelty may be introduced gradually for flexibility training	(11, 18, 26, 52, 53)
Progression rate	Speed and magnitude of increases in intensity, complexity, duration, social demand, or novelty	Weekly increments; task difficulty changes; increase in group size; increased rules or dual-task demands	Progression log; protocolized progression rules; session-by-session adaptation record	Progression is necessary for adaptation but may trigger overload if intensity, complexity, or social/sensory demand increases too quickly	(9, 11, 26, 53)
Environmental adaptation	Degree to which the physical and instructional context is modified for autistic participants	Reduced noise; structured space; visual supports; predictable transitions; adapted equipment; instructor training; home/school/telehealth setting	Environmental checklist; implementation log; instructor training record; caregiver feedback	Determines whether the prescribed dose can be tolerated and delivered consistently in real-world settings	(4, 18, 26, 59, 60, 77, 82, 91)
Adherence burden	Practical, emotional, family, and behavioral cost required to sustain participation	Attendance, dropout, refusal, caregiver burden, travel burden, participant distress, fatigue, adverse events	Attendance logs; dropout reasons; caregiver burden scales; adverse-event log; participant distress monitoring	Converts prescribed dose into delivered and sustained dose; high adherence burden reduces clinical utility even when efficacy is plausible	(4, 18, 26, 52–54, 59, 60, 77, 82)
Safety and adverse-response monitoring	Systematic tracking of physical, behavioral, emotional, and sensory risks during exercise	Injury, excessive fatigue, sleep worsening, behavioral escalation, sensory intolerance, cardiovascular symptoms, dropout due to distress	Adverse-event reporting; caregiver/instructor logs; pre-participation screening; session-level safety monitoring	Necessary because ASD exercise studies often underreport adverse events, null effects, and intolerance; supports balanced clinical translation	(23, 51, 54, 77)
Delivered, tolerated, and sustained dose	Distinction between what was prescribed, what was actually delivered, what the participant tolerated, and what was maintained over time	Prescribed sessions, completed sessions, active participation time, intensity achieved, adaptations required, long-term continuation	Protocol log; attendance record; wearable data; caregiver report; follow-up assessment	Critical in ASD because the same nominal protocol may represent very different effective exposures depending on sensory tolerance, support needs, family context, and motivation	(9, 11, 18, 26, 52, 53, 60, 77, 82, 91)

ASD, autism spectrum disorder; BOT-2, Bruininks-Oseretsky Test of Motor Proficiency, Second Edition; HRR, heart-rate reserve; MABC-2, Movement Assessment Battery for Children, Second Edition; MET, metabolic equivalent; RPE, rating of perceived exertion; TGMD, Test of Gross Motor Development.

#### 3.1.3 From intervention modality to dose structure

A major limitation of existing evidence is its tendency to categorize interventions primarily by type, such as aerobic exercise, martial arts, swimming, horseback riding, exergaming, yoga, or ball sports. Although this approach effectively organizes trials, it lacks conceptual precision. Rather than being a mechanism, modality is a group of interacting requirements. For example, playing a team ball sport requires intermittent physical activity, visual tracking, anticipatory timing, executive switching, shared attention, rule adherence and mutual social interaction. Conversely, water-based exercise changes postural constraints and sensory input, while mind-body exercise may reduce environmental unpredictability and emphasize self-control. Considering these programs merely as ‘types’ of exercise may hide the key elements that truly influence outcomes.

There is growing evidence that the structure of doses could be more closely tied to outcomes than just the modality labels. Group-based organized physical activity has shown favorable effects on social outcomes (36), while mini-basketball training has been associated not only with improved social communication but also with altered white matter integrity, implying that some programs may combine motor, cognitive, and social demands in ways that engage broader neurodevelopmental plasticity (35). Network meta-analysis also suggests that specific structured formats, such as sports games, team ball activities, and combination therapies, might provide comparative benefits for sociability, communication, or executive findings (39, 45). The outcomes should be viewed not as proof that one sport is superior to others, but as an indication that different intervention models activate specific therapeutic pathways.

From a dose-architecture viewpoint, more mechanistically useful questions are posed: How much physiological arousal does the program induce? What degree of cognitive flexibility does the task require? Is it defined by collaborative interaction or primarily by individual repetition? Is the sensory environment stable or disordered? How rapidly does the complexity of a task escalate? These inquiries offer more valuable insights for creating ASD interventions than simply contrasting the effectiveness of ‘basketball’ with ‘swimming’ or ‘yoga’. Reframing interventions in this way also creates a bridge between behavioral outcomes and underlying mechanisms, allowing exercise prescription to be linked more plausibly to executive control, social engagement, arousal regulation, sleep, and sensorimotor adaptation.

#### 3.1.4 The necessity for a narrative review at this time

A narrative review is warranted at this stage because this field is experiencing a phase of accumulating meta-analyses but lacks conceptual cohesion. The literature is now rich in outcome-specific syntheses: there are separate reviews on stereotyped behavior (11, 25), executive functions (8, 45), sleep (20), social communication (28, 38, 39), young adults (6), behavior change techniques (26), exercise prescription (9), and broader clinical trial evidence (5). This expansion is beneficial, yet it has also segmented the field. Although most reviews concentrate on particular questions within distinct fields, there have been few attempts to integrate efficacy, dosage structure, behavioral mechanisms, developmental context, and translational prescription into a single comprehensive framework.

Using a narrative approach is especially warranted since the central scientific challenge extends beyond estimating pooled effect sizes. Rather, this field must explain why similarly labeled interventions produce different outcomes, why some domains improve more reliably than others, why chronic programs may diverge from acute responses, and how exercise can be prescribed in a way that is physiologically meaningful, behaviorally feasible, and developmentally appropriate (6, 12, 22, 23, 27, 29). These integrative questions cannot be answered by meta-analysis alone. A conceptual framework is necessary to link intervention design, participant attributes, potential mechanisms, and real-world application.

**Figure 1:**
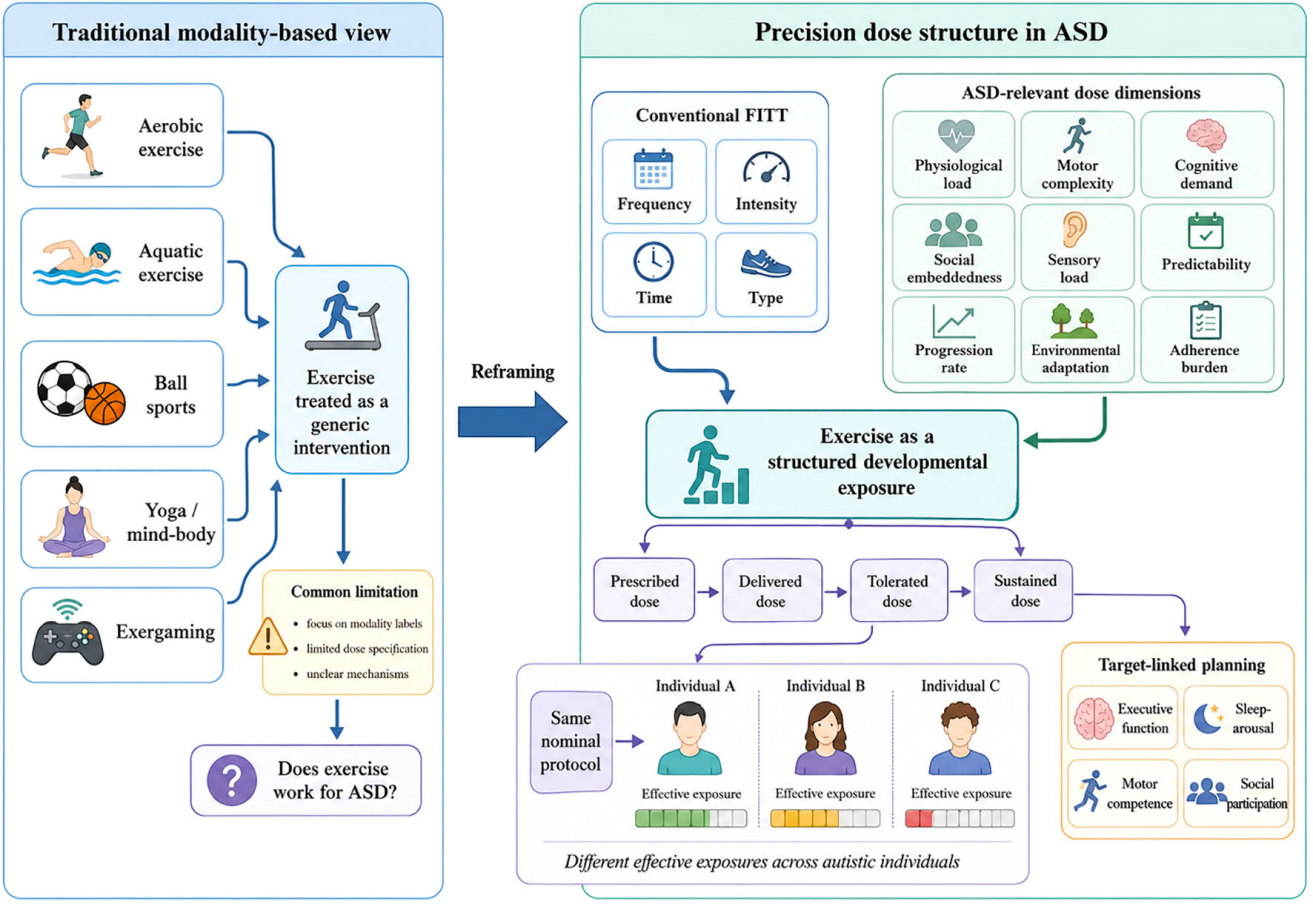
Exercise dose architecture in ASD. The figure decomposes exercise dose into conventional FITT variables and ASD-relevant dimensions, including physiological load, motor complexity, cognitive demand, social embeddedness, sensory load, predictability, progression rate, environmental adaptation, and adherence burden. The figure also distinguishes prescribed dose, delivered dose, tolerated dose, and sustained dose, emphasizing that the same nominal protocol may represent different effective exposures across autistic individuals.

Therefore, the purpose of this review is not to re-examine whether exercise is generally beneficial for ASD, but to refocus this field on a more practical question: how exercise dose can be interpreted, mechanistically explained, and clinically recommended as a precise developmental intervention for ASD. Within this model, exercise is not merely seen as a typical supportive activity, but as a structured intervention whose effectiveness is contingent upon the dosage design, timing of execution, fit with the participant’s environment, and particular outcome system being targeted. Supported by scientific research, this novel perspective is vital for clinical practice. **Figure 1** shows the reframing exercise for autism spectrum disorder, moving from a modality-based approach to a precision dose structure.

### 3.2 Mechanisms by which exercise dose might affect ASD

Current understanding of exercise effects in ASD remains limited, with most evidence focusing on behavioral changes, peripheral biomarkers, and a small number of neuroimaging studies. Even so, recent ASD-specific reviews and broader developmental-disability syntheses converge on several recurring candidate pathways, including neuroplasticity and synaptic remodeling, arousal and sleep regulation, neuroimmune modulation, gut-brain signaling, and cognitive-motor coupling (14, 46). Importantly, the available literature also suggests that chronic exercise exposure is more likely than single-bout exercise to induce durable neural or behavioral adaptation, which is consistent with the idea that exercise dose in ASD should be interpreted as a repeated biological signal instead of a one-time stimulus (14, 27, 46). Importantly, the following mechanisms should not be interpreted as equally established. Sleep-related outcomes and selected behavioral effects have relatively stronger ASD-specific clinical support, whereas neuroimmune, gut-brain, and some neuroimaging pathways remain candidate mechanisms requiring direct mediation tests in ASD exercise trials. **Table 3** summarizes the major candidate mechanisms, the dose-architecture dimensions most likely to engage each pathway, the directness of supporting evidence, and the key research gaps that should be addressed before these mechanisms can be used for precision exercise prescription in ASD.

**Table 3:** Candidate mechanisms linking exercise dose architecture to ASD-relevant outcomes: evidence level and research gaps.

Candidate mechanism	Dose-architecture dimensions most relevant	ASD-specific clinical/behavioral evidence	ASD-specific biomarker, physiological, or neuroimaging evidence	Animal-model or indirect evidence	Candidate biomarkers/readouts	Evidence level	Main research gaps	Literature support
Neuroplasticity, synaptic remodeling, and network adaptation	Repeated exposure; intervention duration; motor complexity; cognitive demand; progression rate	Exercise interventions have been associated with improvements in executive function, social communication, and core symptoms in ASD, particularly in chronic programs rather than isolated acute bouts	Mini-basketball training was associated with improved social communication and altered white matter integrity; aquatic exercise was associated with improved executive function and increased BDNF levels; group basketball-based motor skill learning was associated with improved joint attention and white matter changes	Broader exercise neuroscience supports exercise effects on BDNF, synaptic plasticity, learning-related adaptation, and network remodeling; animal models suggest exercise may increase hippocampal BDNF and improve cognition	BDNF; white matter integrity; executive-function tasks; joint attention; social communication measures; neuroimaging markers	Level B for ASD-specific human mechanistic evidence; Level C for animal and broader exercise neuroscience	Few ASD trials directly test mediation; limited sample sizes; sex imbalance; unclear dose-response relationship between motor/cognitive complexity and neural adaptation	(7, 12, 33–35, 45–48)
Arousal regulation, sleep stabilization, and circadian rhythm	Physiological load; exercise timing; frequency; predictability; intervention duration; sensory load	Physical activity interventions have shown benefits for sleep quality and sleep-related outcomes in children and adolescents with ASD	Morning jogging improved sleep efficiency, wake after sleep onset, and melatonin levels; water-based exercise improved sleep quality; cycling and melatonin showed comparable sleep-related effects in one trial	Broader chronobiology literature supports exercise as a non-photic regulator of sleep-wake physiology, but ASD-specific circadian-marker evidence remains limited	Sleep efficiency; wake after sleep onset; sleep onset latency; actigraphy; sleep diary; melatonin; daytime behavior	Level A for ASD-specific sleep outcomes; Level B for melatonin/actigraphy-supported ASD evidence	Need trials comparing morning vs afternoon/evening exercise; limited circadian phase markers; unclear optimal intensity and timing for sleep benefits	(20, 49–52)
Neuroinflammation and immunometabolic modulation	Physiological load; intervention duration; recovery; safety monitoring; progression rate	Some ASD exercise studies report behavioral or sleep improvements that may align with inflammatory changes, but clinical evidence remains limited	Water-based exercise reduced serum IL-1β and TNF-α in boys with ASD while improving sleep quality	Valproic-acid animal models suggest swimming or treadmill exercise may reduce neuroinflammation and improve behavioral or cognitive outcomes	IL-1β; TNF-α; cytokine panels; oxidative-stress markers; sleep and behavior outcomes	Level B for limited ASD peripheral biomarker evidence; Level C for animal-model evidence	Peripheral cytokines may not reflect central neuroinflammation; few dose-stratified trials; unclear whether inflammatory changes mediate behavioral improvement	(12, 46, 51, 53)
Oxidative stress and immunometabolic stress regulation	Physiological load; intervention duration; progression rate; recovery; participant health status	Direct ASD clinical exercise evidence remains sparse	Limited ASD-specific biomarker evidence; oxidative stress is often discussed together with inflammatory and metabolic pathways	Broader ASD and exercise literature supports biological plausibility, but direct exercise-dose evidence in ASD is weak	Oxidative-stress markers; antioxidant capacity; inflammatory markers; metabolic profile; fatigue	Level C–D	Need ASD-specific trials measuring oxidative-stress markers before and after defined exercise doses; unclear clinical relevance and mediation	(12, 46, 53)
Gut-brain interactions and microbiota-related metabolic signaling	Intervention duration; physiological load; regularity; diet context; adherence; environmental stability	Direct human ASD exercise trials linking microbiota change to symptom improvement are currently limited	ASD-specific human exercise-microbiome evidence remains insufficient for causal inference	A valproic-acid autism-like rat model showed that voluntary exercise altered gut microbiota, short-chain fatty acids, neurotransmitter-related metabolites, and behavior; fecal microbiota transplantation reproduced some benefits	Gut microbiota composition; short-chain fatty acids; metabolomics; gastrointestinal symptoms; behavioral outcomes	Level C for animal-model causal evidence; Level D for human ASD clinical translation	Need human ASD trials integrating exercise dose, diet, microbiome, metabolomics, GI symptoms, and behavioral outcomes; causality remains unproven in humans	(13, 54, 55)
Cognitive-motor coupling and sensorimotor integration	Motor complexity; cognitive demand; task sequencing; object control; social embeddedness; progression rate	Mini-basketball and coordinative exercise interventions have been associated with improvements in executive function, joint attention, social communication, and core symptoms	Group motor learning and basketball-based interventions have been associated with white matter changes relevant to attention, sensory processing, and social-motor function	Broader ASD literature links motor skills with social communication, language, adaptive function, and attention	Fundamental motor skills; balance; object control; executive function; joint attention; social communication; white matter integrity	Level B for ASD-specific intervention plus neuroimaging evidence; Level A/B for behavioral outcomes depending on domain	Need dismantling studies to separate motor complexity, social interaction, and aerobic intensity; limited evidence on which motor components drive social or cognitive gains	(7, 30, 34, 35, 48, 56, 73)
Social engagement, shared attention, and participation pathways	Social embeddedness; group structure; cooperation vs competition; predictability; sensory load; adherence burden	Group-based physical activity, mini-basketball, sports games, and structured physical activity have been associated with improvements in social interaction, communication, and participation	Some basketball-based studies report white matter or joint-attention-related changes, but biomarker evidence remains limited	Indirect evidence from developmental and motor-social literature suggests shared movement may support attention, imitation, timing, and interpersonal coordination	Social communication scales; joint attention; participation measures; peer interaction; caregiver report	Level A for selected social outcome meta-analytic evidence; Level B/C for mechanism-specific evidence	Need to distinguish therapeutic social engagement from social overload; little evidence on females, masking burden, anxiety, or sensory intolerance during group exercise	(24, 33–37, 48, 56)
Sensory regulation and environmental fit	Sensory load; predictability; environmental adaptation; transition demands; adherence burden	Aquatic therapy and adapted physical activity may improve participation, social competence, quality of life, or tolerance in some autistic individuals	Direct sensory biomarkers are rarely measured in ASD exercise trials	Broader ASD sensory-processing literature supports the importance of sensory adaptation for participation and behavioral regulation	Sensory profile; distress/refusal logs; participation; quality of life; adherence; transition tolerance	Level C–D	Need standardized sensory-load coding; unclear which sensory environments improve tolerance versus trigger dysregulation; limited adverse-response reporting	(11, 18, 38, 39, 79)
Adherence, feasibility, and implementation mechanisms	Adherence burden; environmental adaptation; caregiver involvement; telehealth delivery; preference; progression rate	Parent-mediated, family-based, telehealth-supported, and digital interventions show feasibility and potential benefits for physical activity, motor skills, sleep, or participation	Mechanistic biological evidence is generally not the focus of implementation studies	Implementation science and behavior-change literature support structured monitoring of reach, retention, caregiver burden, and sustainability	Attendance; dropout; caregiver burden; adverse events; physical activity monitoring; retention; acceptability; implementation cost	Level B/C for feasibility and implementation evidence	Need long-term pragmatic trials; poor reporting of adverse events and dropout reasons; limited evidence on sustained behavior change after intervention ends	(4, 18, 26, 59, 60, 77, 78, 82, 91)

Evidence level was classified as follows: Level A, ASD-specific meta-analysis or randomized controlled evidence for a clinical or behavioral outcome; Level B, ASD-specific human biomarker, neuroimaging, physiological, or mechanism-linked intervention evidence; Level C, ASD animal-model evidence, non-ASD human exercise evidence, or indirect developmental evidence; Level D, theoretical or hypothesis-generating extrapolation.

#### 3.2.1 Neuroplasticity, synaptic remodeling, and network adaptation

One candidate explanation is that repeated exercise may support neuroplasticity-related processes (14, 47–49), but the strength of evidence differs across levels: broader exercise neuroscience and animal-model studies provide biological plausibility (47–49), whereas ASD-specific human evidence remains limited to a smaller number of biomarker and neuroimaging studies (15, 36, 50). In the broader exercise neuroscience literature, exercise is strongly linked to brain-derived neurotrophic factor (BDNF) signaling and to the modulation of synaptic plasticity, including processes relevant to long-term potentiation, network refinement, and learning-dependent adaptation (47, 48). ASD-specific evidence is now beginning to align with this framework. In children with ASD, a 12-week aquatic exercise intervention improved inhibition control and cognitive flexibility while also increasing BDNF levels, revealing that at least some cognitive benefits may be accompanied by physiologically meaningful shifts in trophic signaling (33). Similarly, a recent review emphasized that neuronal growth factors and signaling related to plasticity are central mediators in the mechanisms of exercise for autistic individuals, rather than peripheral elements (12).

In ASD, neuroplasticity should be examined at the systems level in addition to the level of circulating biomarkers. A mini-basketball training program was reported to improve social communication together with white matter integrity in children with autism, providing one of the clearest examples that exercise-related behavioral change may be accompanied by measurable structural brain adaptation (35). More recently, group basketball-based motor skill learning was shown to improve joint attention, and these changes were associated with white matter alterations in tracts related to sensory processing, spatial attention, and early attentional orienting (48). In parallel, animal work has strengthened the biological plausibility of this pathway: adolescent treadmill exercise in autism-modeled rats increased hippocampal BDNF expression and improved cognition, supporting the idea that repeated exercise can shape plasticity-related neural substrates during development (47). Overall, these results propose a model in which exercise levels may influence ASD by not only ‘increasing activity’ but also by progressively changing neural systems associated with attention, learning, communication, and adaptive behavior (15, 49, 50).

#### 3.2.2 Arousal regulation, sleep, and circadian stabilization

Another key pathway involves the control of wakefulness and sleep. In ASD, sleep issues are widespread and closely tied to daytime behavior, emotional instability, and family stress, underscoring their significance as a clinical and mechanistic area of interest (20). A recent systematic review and meta-analysis concluded that physical activity interventions can improve sleep in children and adolescents with ASD, while intervention studies have shown that exercise may enhance sleep efficiency, reduce wake after sleep onset, and improve overall sleep quality (9, 51–53). Significantly, in children with ASD, morning jogging is associated with improved sleep and higher endogenous melatonin levels, whereas aquatic exercises are also connected to better sleep patterns (51, 52). According to these findings, exercise may help individuals with ASD by balancing physiological arousal and sleep-wake cycles, rather than directly modifying core social symptoms (9, 51–53).

In ASD exercise research, the circadian aspect is conceptually important but lacks empirical maturity. In the broader chronobiology literature, exercise is increasingly viewed as a non-photic zeitgeber capable of influencing circadian phase, melatonin dynamics, and sleep-related physiology, with timing and intensity helping determine the direction and magnitude of these effects (47, 54). In ASD, most intervention studies have focused on measuring sleep outcomes instead of directly assessing circadian phase markers. This reveals that circadian stabilization is more of a plausible mechanistic extension at present, instead of a completely confirmed pathway specific to ASD. However, the discovery that exercise may improve sleep in children with ASD by influencing melatonin levels indicates that circadian regulation could be a valuable focus for future dose-response studies (47, 51, 53, 54).

#### 3.2.3 Neuroinflammation, oxidative stress, and immunometabolic modulation

Neuroimmune and inflammatory pathways represent a plausible but still incompletely established mechanism through which exercise may influence ASD-related biology (14, 48, 52, 55). Reviews of the ASD literature have repeatedly highlighted altered inflammatory profiles, glial activation, and cytokine dysregulation as candidate contributors to symptom expression and developmental vulnerability, and exercise has been proposed as a non-pharmacological means of modulating this inflammatory milieu (46). In this framework, exercise is not merely viewed as improving behavior from the outside; rather, it may act by shifting neuroimmune tone, reducing pro-inflammatory signaling, and indirectly restoring a more permissive environment for neural adaptation and behavioral regulation (14, 48).

The clearest ASD-linked evidence comes from intervention and animal-model studies. For children with ASD, taking part in water-based exercise improved sleep quality and decreased serum IL-1β and TNF-α levels, suggesting that behavioral improvements may align with observable anti-inflammatory effects (51). Swimming exercise during adolescence in a prenatal valproic acid model boosted cognitive function, mitigated stress symptoms, and lowered neuroinflammation, with brain cytokines influencing these effects (53). Although direct evidence specifically linking exercise dose to oxidative stress trajectories in ASD remains limited, current reviews consistently place oxidative stress within the same mechanistic cluster as neuroinflammation and immunometabolic dysregulation (14, 48). Thus, the most defensible interpretation at present is that exercise in ASD may partly work by rebalancing inflammatory and metabolic stress systems, but this field still lacks sufficiently detailed dose-stratified biomarker studies to define which programs are anti-inflammatory, for whom, and under what developmental conditions (48, 52, 55).

#### 3.2.4 Gut-brain interactions and exercise-responsive metabolic signaling

Gut-brain signaling is an emerging candidate pathway in ASD exercise research, but direct human evidence linking exercise dose, microbiota change, and clinical improvement in autistic individuals remains limited (16, 56, 57). Reviews on ASD have recently suggested that modifications in gut microbial ecology, microbial metabolites, and neuroimmune signaling caused by exercise might partly contribute to the clinical benefits of physical activity (16, 56). The argument is bolstered by the frequent correlation of ASD with gastrointestinal issues and changes in microbiota profiles, revealing that gut-responsive mechanisms are more than just speculative (54). In this context, not only might exercise function as a neural intervention, but it could also impact metabolism and microbes, altering the biological underpinnings of behavior (16, 56).

Gut-brain signaling is an emerging candidate pathway in ASD exercise research, but direct human evidence linking exercise dose, microbiota change, and animal-model evidence has strengthened the biological plausibility of this pathway, but it should not be interpreted as direct clinical evidence in autistic humans (55). A 2026 study in a valproic acid-induced autism-like rat model showed that six weeks of voluntary exercise alleviated autism-like behaviors, altered gut microbiota composition, and modulated short-chain fatty acids and neurotransmitter-related metabolic readouts; notably, fecal microbiota transplantation from exercised animals reproduced behavioral and metabolic benefits in recipient animals (55). This finding is important because it shifts the gut pathway from mere correlation to a more causally informed mediation, although clinical translation is still in its early stages. There are limited studies that integrate exercise dose architecture, gut microbiota analysis, metabolomics, dietary habits, and symptom progression in humans with ASD. Hence, the gut-brain mechanism holds great promise, yet it lacks the detailed specification needed for precision-informed prescriptions (16, 56, 57).

#### 3.2.5 Cognitive-motor coupling, sensorimotor integration, and social function

A crucial pathway in ASD could be the link between cognitive and motor functions. In ASD, motor differences often arise and can be observed early in development, with increasing evidence showing that these skills are not isolated deficits but are related to language, social communication, attention, and overall adaptive functioning (5, 58). A comprehensive review on the relationship between social and motor skills in ASD found that these aspects are frequently interrelated, with object control and manual dexterity playing significant roles (56). This is highly consequential for exercise science, because it implies that movement-based interventions may influence social and cognitive outcomes not only through physiological load, but also through improvements in action planning, imitation, shared attention, timing, and sensorimotor prediction (5, 58).

This interpretation is backed by intervention studies. The mini-basketball training literature has shown improvements in executive functions and core symptoms in preschool children with ASD (34), while later studies linked similar group motor learning formats to better joint attention and white matter adaptation (48). According to the results, exercise routines that involve intricate coordination, perception, and interaction could more effectively trigger mechanisms pertinent to ASD compared to those that emphasize just aerobic intensity. To put it differently, exercise may benefit children by helping them connect perception with action, action with concentration, and concentration with socially significant goals. This makes sensorimotor integration a particularly important bridge between physical dose and social function, and it further supports the view that exercise modality should be decomposed into mechanistic ingredients such as motor complexity, shared task structure, and attentional demand (5, 37, 50, 58).

**Figure 2:**
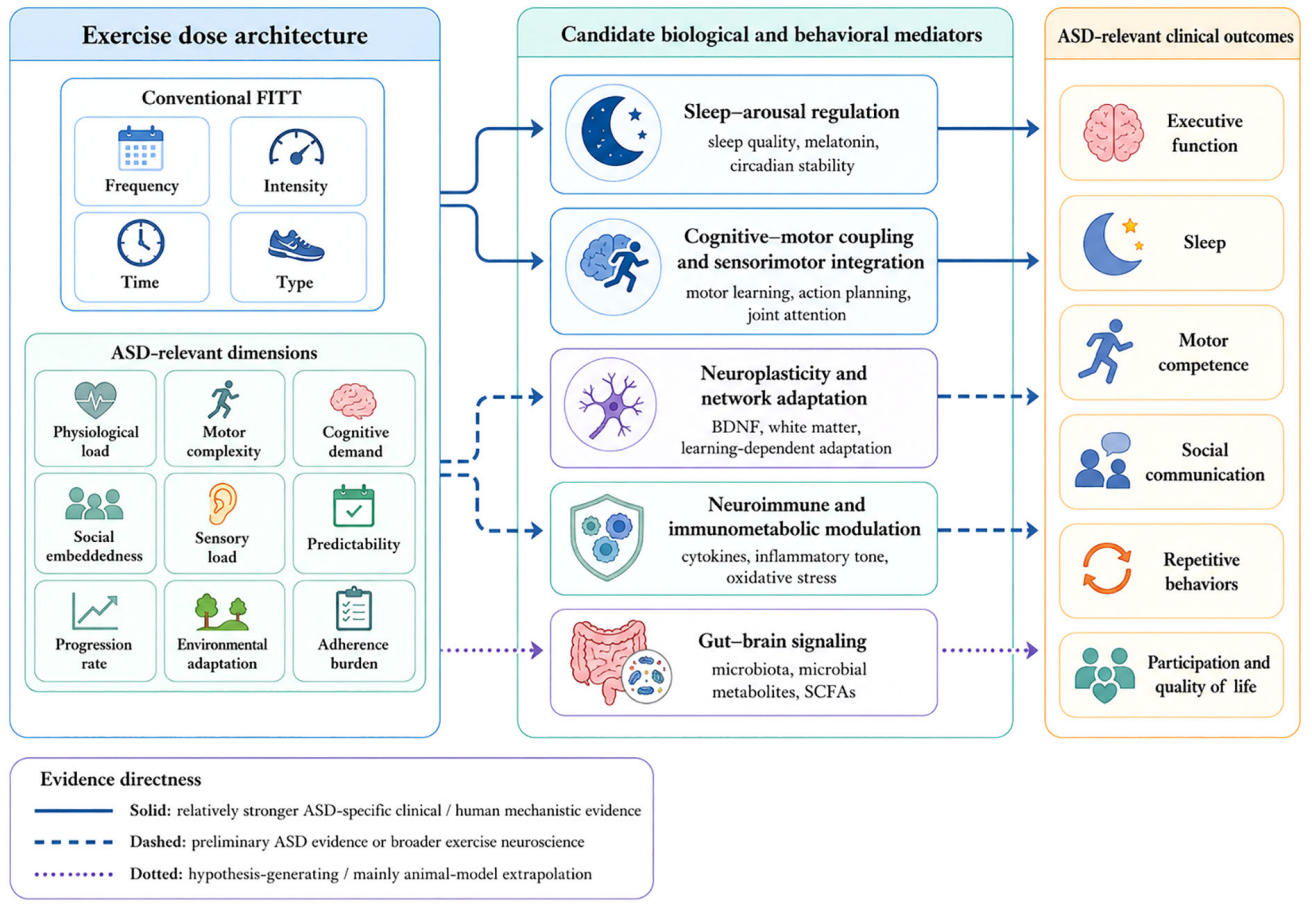
Mechanistic pathways are displayed according to evidence directness. Solid lines indicate pathways with relatively stronger ASD-specific clinical or human mechanistic evidence, dashed lines indicate pathways supported mainly by ASD-specific preliminary studies or broader exercise neuroscience, and dotted lines indicate pathways that remain largely hypothesis-generating or extrapolated from animal models. The figure distinguishes clinical outcomes from candidate biological mediators to avoid implying that all mechanisms are established causal pathways in ASD.

Existing research generally supports a model with multiple pathways, suggesting that exercise dose could impact ASD via somewhat overlapping biological and behavioral systems instead of one unified mechanism. The strongest ASD-linked evidence currently supports neuroplasticity-related adaptation, sleep-arousal regulation, neuroimmune modulation, gut-responsive signaling, and cognitive-motor coupling, but the relative contribution of each pathway likely varies across individuals, developmental stages, and exercise architectures (14, 16, 46, 48). The range of differences is exactly why future investigations should extend beyond the usual assertions that ‘exercise is beneficial’ and pinpoint which exercise doses specifically engage which mechanisms in distinct ASD subgroups (14, 46). **Figure 2** illustrates the mechanisms by which exercise dose might affect biology and behavior related to ASD.

### 3.3 Sex- and development-related heterogeneity: evidence gaps and research priorities

In this review, biological sex refers to male/female classification and puberty-related biological development, whereas gender-related constructs refer to social experience, identity, masking or camouflaging, diagnostic bias, and gendered participation barriers. We therefore use “sex-related” when referring to biological or pubertal factors and “gender-related” when referring to social or diagnostic constructs. Sex- and development-related variability is an important but under-tested issue in ASD exercise research (1, 3, 6, 17, 18, 59, 60). Current exercise-specific evidence is insufficient to support firm sex-specific exercise prescriptions (6, 61, 62), but broader ASD research on female underrepresentation, diagnostic bias, pubertal development, and camouflaging strongly supports the need for sex-aware recruitment, reporting, and analysis (1, 3, 17–19, 59, 60). Although physical activity and exercise interventions are increasingly studied in ASD, most syntheses still pool participants across broad developmental ranges, rarely report sex-stratified analyses, and remain heavily weighted toward child and adolescent samples. Reviews focused on young adults emphasize that adult evidence is still sparse, while adult studies on participation barriers and telehealth-based activity support remain largely feasibility-oriented instead of mechanistically or sex-stratified designed (6, 61, 62). This problem sits within a broader autism literature that has long been male-skewed, from prevalence estimates and diagnostic pathways to neurobiological research samples, thereby constraining the interpretability and generalizability of exercise-response models in girls and women (1, 3, 59, 63).

#### 3.3.1 Female underrepresentation and the limits of the current evidence base

The underrepresentation of females in ASD exercise studies is not just about sample imbalance; it also concerns case identification. The historical male-to-female ratio in autism has often been interpreted as reflecting biological liability, but meta-analytic and recent review evidence also indicates substantial diagnostic bias, with girls more likely to be missed, diagnosed later, or identified only when symptom burden is more obvious or accompanied by co-occurring difficulties (1, 3, 59). This directly affects this field of exercise science. If girls and women are less likely to be identified unless they present with more overt or impairing profiles, then female participants enrolled into ASD intervention studies may represent a selectively recognized subgroup instead of the full female autism phenotype (3, 59).

In exercise research, this limitation is heightened by the limited demographic range of existing studies. The majority of intervention studies concentrate mainly on boys in their childhood, whereas studies involving adults are less common and often highlight participation, feasibility, or altered delivery methods rather than specific guidelines (6, 61, 62). Research on physical activity among autistic adults reveals low engagement levels and important social, sensory, and logistical challenges (59), whereas telehealth and peer-supported approaches appear feasible but have not yet demonstrated sex-specific outcomes (60). Consequently, current evidence is not robust enough to ascertain whether males and females with ASD react differently to exercise; it lacks the structure needed to confidently pinpoint such differences (6, 61, 62).

Where sex distribution was available in the reviewed ASD exercise literature, the samples were predominantly male. However, because sex-specific reporting was inconsistent across studies and reviews, the current literature does not allow a precise pooled estimate of female representation without a formal systematic re-analysis. We therefore summarize female proportion at the study level in **Table 1** and interpret all sex-specific implications as research priorities rather than established clinical recommendations.

#### 3.3.2 Early phenotypic similarity and later divergence in developmental trajectories

Increasing developmental research indicates that sex-related differences in ASD might not be accurately captured by a straightforward early binary division. An extensive study conducted recently on young children indicated that there are little to no phenotypic sex differences when toddlers with ASD begin to show symptoms (14). This aligns with reviews centered on lifespan, revealing that sex differences might become more apparent as development progresses, instead of being completely obvious from the earliest stage (58). Simultaneously, research indicates that females with autism exhibit phenotypic differences from males that are often subtle, partly qualitative, and not always captured by standard screening techniques (63). Overall, these findings suggest that the initial resemblances between males and females might eventually diverge into differences as social pressures, coping methods, concurrent conditions, and diagnostic processes become more significant during childhood and adolescence (17, 60, 64).

This viewpoint on development is very pertinent to the study of exercise. If sex differences are relatively muted at first symptom onset but become more apparent later, then observed differences in adolescent or adult exercise studies may reflect cumulative developmental divergence instead of immutable biological separation from the start. The model aligns with data on delayed diagnosis, suggesting that even when early symptoms are similar or slightly different, girls may not be diagnosed until a later age. This implies that the factors influencing study participants and the timing of their enrollment include not only the initial phenotype but also developmental trajectories (21, 65). Therefore, differences in exercise response linked to sex should be regarded as being at least partly dependent on trajectory. During an ASD exercise trial, female adolescents or adults may vary from their male peers not only in terms of gender but also in the duration of recognition, social adaptation, internalized issues, and availability of intervention opportunities (21, 65).

#### 3.3.3 Puberty, hormonal transition, and age-sensitive dose responsiveness

One of the key but frequently neglected factors impacting exercise response in ASD is probably puberty. Studies, both cross-sectional and longitudinal, have noted differences in pubertal development related to ASD. Research found that females with ASD began puberty sooner than both males with ASD and typically developing females, including an earlier onset of menstruation (65). In a later longitudinal study, both male and female ASD youth experienced earlier onset of puberty, with males typically progressing through puberty more quickly than females (16). Studies conducted recently have supported the idea that puberty in those with autism might progress in unusual or inconsistent ways, with the specific pattern differing according to the sample and measurement techniques (66). These findings matter because puberty is not simply a hormonal milestone; it is a period of major reorganization in sleep, emotion regulation, body awareness, social salience, and behavioral tolerance to stressors, all of which may alter how a given exercise dose is experienced and sustained (18, 66, 67).

Nevertheless, studies on exercise in ASD rarely categorize participants according to their pubertal stage. Numerous meta-analyses combine children and adolescents across wide age spans, while studies on young adults are typically treated as separate literature rather than as continuations of evolving response trends (4, 6, 8). This implies that the responsiveness to doses according to age has not been extensively tested. The same nominal intervention intensity may have very different effects before, during, and after puberty because the relevant mediators—sleep stability, internalizing symptoms, social self-consciousness, sensory sensitivity, fatigue, and adherence—are themselves developmentally dynamic (4, 8, 18, 66–68). Therefore, future ASD exercise trials should not rely on chronological age alone. Adolescent samples should document pubertal stage using feasible measures such as Tanner staging, the Pubertal Development Scale, age at menarche where relevant, growth-spurt timing, and puberty-related changes in sleep, mood, fatigue, body awareness, and exercise tolerance.

#### 3.3.4 Sex-related phenotype expression, masking, and outcome measurement

The expression of sex-related phenotypes adds complexity to understanding the effects of exercise in ASD. Reviews and first-person studies of autistic girls and women repeatedly highlight that female presentation may include less obvious or more socially masked autistic traits, more context-dependent social adaptation, and restricted interests that appear more socially acceptable than the stereotyped interests often emphasized in male-oriented descriptions (19, 64, 69–71). Systematic reviews of camouflaging show that social masking is a central construct in autism research and is often reported more prominently in females, while adult-focused reviews indicate that camouflaging can obscure symptom visibility in exactly those contexts where standard observational metrics are expected to perform best (19, 69). Evaluations of measurements reveal ongoing disagreements about the best way to define social camouflaging, leading to challenges in both methodology and clinical practice (70).

These problems directly impact exercise trials. If outcome batteries are centered on overt social withdrawal, externally visible repetitive behavior, or broad symptom totals, they may miss clinically relevant changes in autistic girls and women whose major burden lies in internalizing symptoms, effortful compensation, sensory overload, or exhaustion from social masking (19, 20, 64, 68–71). This issue is not theoretical. Educator-based research suggests that girls are less readily recognized as autistic in naturalistic settings (15), and studies of adolescence indicate that females with ASD may show heightened depressive or internalizing vulnerability at specific developmental stages (68, 72). As a result, an intervention might deliver crucial advantages—such as decreased social fatigue, enhanced self-control, or increased participation—without causing substantial changes in typical male-oriented or externally focused ASD outcomes. Research on exercise in ASD needs not only balanced sampling of sexes but also outcome measurements that are sensitive to sex-related phenotype expression and the burden of masking (19, 20, 69–71).

#### 3.3.5 Toward a sex-informed and stage-informed response model

A more robust approach in this field is to prevent assuming inherent male-female differences in exercise effectiveness, and instead reveal a model that considers sex and developmental stages. According to this model, the exercise response is determined by at least five interacting components: developmental stage, changes during puberty, expression of sex-related characteristics, concurrent internalizing issues, and access to continuous participation opportunities (4, 7, 8, 17, 18, 21, 60, 64–68, 73). Compared to a basic binary comparison, this method better aligns with existing studies by taking into account two important aspects: initially, early phenotypic sex differences might not be as crucial as previously assumed; Secondly, later developmental differences, masking, and diagnostic selection can significantly change who participates in ASD exercise studies and how the effects of interventions are observed (17, 19–21, 60, 64, 65, 69–71).

Within this framework, future research should systematically document gender-specific recruitment, retention, and adherence rates; focus on adolescence or developmental stages rather than age alone; and present findings that go beyond mere relief of visible symptoms to include the quality of participation, internalizing symptoms, the burden of masked symptoms, self-assessed social functioning, and participation in physical activity in real-life settings (4, 7, 8, 19, 61, 62, 68, 69, 73). The main takeaway is that precision exercise for ASD involves more than just optimizing the physiological dose. It will require recognizing that the same exercise architecture may impose different social, sensory, and emotional loads depending on whether the participant is a prepubertal boy, an adolescent girl navigating masking demands, or an autistic young adult with longstanding participation barriers. Without trials designed with such detailed stratification, the lack of uncovered sex differences should be viewed cautiously as a sign of inadequate resolution, instead of true equality (4, 6–8, 19, 20, 61, 62, 68–71, 73).

### 3.4 Clinical translation: from efficacy studies to precision exercise prescriptions

To make the framework clinically interpretable, we propose a stepwise decision model rather than a single universal exercise prescription. The model begins with the primary functional target, then evaluates participant characteristics, selects an appropriate dose architecture, chooses a delivery context, monitors safety and adherence, and adjusts the program according to response. Step 1 is to define the primary target domain, such as sleep, executive function, motor competence, social participation, repetitive behavior, anxiety-related avoidance, or quality of life (4, 8, 9, 11, 12, 39, 74, 75). Step 2 is to characterize the participant profile, including age, pubertal stage, sex, support needs, intellectual and adaptive functioning, baseline motor competence, sensory profile, sleep/arousal pattern, co-occurring ADHD or anxiety, medical risks, and family resources (1, 3, 5, 7, 17–19, 22, 40, 41, 59, 60, 68, 76–78). Step 3 is to select the dose architecture, including physiological load, motor complexity, cognitive demand, social embeddedness, sensory load, predictability, and progression rate (12, 13, 22, 23, 42, 43). Step 4 is to choose the delivery context, such as home, school, clinic, community, caregiver-mediated, peer-supported, or telehealth-supported delivery (7, 22, 23, 61, 62, 79–82). Step 5 is to monitor attendance, perceived exertion, distress, adverse events, sleep change, behavioral dysregulation, and caregiver burden (9, 22, 23, 31, 44, 51, 52, 79). Step 6 is to adjust the program by increasing complexity only after tolerance is established, reducing sensory or social load when dysregulation occurs, and modifying timing or intensity when sleep or arousal is the primary target (9, 12, 13, 22, 23, 40, 41, 51, 53, 83). **Table 4** outlines Clinical decision model for matching exercise dose architecture to ASD-related functional targets.

**Table 4:** Clinical decision model for matching exercise dose architecture to ASD-related functional targets.

Primary functional target	participant characteristics to assess	Dose-architecture emphasis	Example exercise modalities or delivery formats	Monitoring indicators	Cautions and adjustment rules	Literature support
Sleep stabilization and arousal regulation	Sleep disturbance; bedtime routine; daytime fatigue; sensory sensitivity; exercise timing tolerance; medication use; family schedule	Moderate physiological load; high predictability; consistent timing; low-to-moderate sensory load; gradual progression	Morning jogging; cycling; aquatic exercise; structured aerobic routines; caregiver-supported home exercise	Sleep efficiency; wake after sleep onset; sleep onset latency; sleep diary; actigraphy; melatonin where feasible; daytime behavior	Avoid overstimulating sessions close to bedtime; reduce intensity or sensory/social load if sleep worsens; prioritize routine and timing consistency before increasing complexity	(20, 49–52)
Executive function and cognitive control	Baseline inhibition, cognitive flexibility, attention, working memory, ADHD symptoms, task tolerance, motor planning ability	Moderate-to-high cognitive demand; motor complexity; rule switching; inhibition requirements; progressive task difficulty	Mini-basketball; coordinative exercise; ball games; exergaming; structured motor-skill training; dual-task movement games	Executive-function tasks; inhibition and cognitive-flexibility measures; BRIEF or caregiver-rated executive function; task performance; engagement	Do not increase cognitive complexity before basic task tolerance is established; reduce rule-switching demands if frustration or refusal emerges	(7, 33, 34, 36, 43, 73)
Motor competence and sensorimotor integration	Baseline motor delay; balance; bilateral coordination; object-control skills; postural control; imitation ability; motor confidence	High motor-complexity specificity; progressive motor sequencing; object control; balance challenge; repetition with structured feedback	Fundamental motor skill training; balance training; aquatic therapy; ball skills; martial arts; structured motor learning	TGMD, BOT-2, MABC-2, balance tests, object-control performance, participation quality	Match task difficulty to motor baseline; avoid rapid progression that increases failure experience; prioritize mastery and confidence	(11, 30, 34, 35, 48, 56, 77, 82)
Social communication and social participation	Social motivation; peer tolerance; masking burden; anxiety; sensory tolerance in group settings; communication level; preference for individual vs group activity	Social embeddedness; shared attention; cooperative structure; predictable peer interaction; moderate motor and cognitive demand	Cooperative ball games; mini-basketball; group motor skill learning; peer-assisted activity; caregiver-mediated social play; structured team games	Social communication scales; joint attention; peer interaction; participation frequency; caregiver/instructor report; distress during group activity	Distinguish therapeutic social exposure from social overload; use dyadic or small-group formats before competitive team formats when tolerance is low	(24, 33–37, 48, 56, 60)
Repetitive behavior and behavioral self-regulation	Type and context of repetitive behavior; arousal pattern; anxiety; sensory triggers; transition difficulty; behavioral function of movement	Predictable structure; rhythmic or repetitive motor routines; moderate physiological load; low uncertainty; gradual novelty	Jogging; cycling; swimming; martial arts; yoga; structured aerobic routines; predictable motor circuits	Repetitive behavior scales; behavioral observation; distress/refusal log; transition tolerance; caregiver report	Do not use exercise as punishment or suppression; monitor whether high-intensity or chaotic contexts increase stereotypy or distress	(3, 8, 10, 21, 23)
Anxiety-related avoidance and emotional regulation	Anxiety symptoms; avoidance patterns; social fear; sensory intolerance; masking burden; co-occurring internalizing symptoms; prior negative sport experiences	Low initial social and sensory load; high predictability; participant choice; gradual exposure; autonomy-supportive progression	Individual walking or cycling; yoga; aquatic exercise; home-based exercise; telehealth-supported activity; preferred leisure activity	Anxiety symptoms; refusal/avoidance; distress rating; attendance; perceived control; caregiver burden	Avoid forcing group or competitive formats too early; prioritize perceived safety, preference, and predictability before increasing social exposure	(18, 59–61, 67, 89)
Quality of life, participation, and family functioning	Family schedule; caregiver capacity; access barriers; participant preference; transportation; cost; school/community resources; long-term adherence	Low adherence burden; high environmental fit; flexible delivery; family or caregiver involvement; sustainable intensity	Family-based physical activity; leisure programs; aquatic therapy; caregiver-mediated programs; community-based adapted exercise; telehealth-supported programs	Quality of life; participation frequency; attendance; caregiver burden; satisfaction; retention; physical activity level	The most effective protocol is clinically weak if it cannot be sustained; adapt delivery context before escalating dose	(4, 18, 26, 59, 60, 77–80, 82, 91)
Physical fitness and metabolic health	Baseline fitness; obesity or metabolic risk; sedentary behavior; motor limitations; cardiovascular tolerance; medical clearance needs	Physiological load; frequency; session duration; progressive overload; long-term adherence	Aerobic exercise; cycling; walking/jogging; swimming; circuit training; combined aerobic and resistance training	Heart rate; RPE; accelerometry; MVPA; BMI or body composition where relevant; fitness tests; adverse events	Increase intensity gradually; monitor fatigue, injury, cardiovascular symptoms, and dropout; avoid making fitness goals override sensory or emotional tolerance	(6, 9, 11, 74, 81, 91)
Real-world physical activity engagement	Motivation; preference; autonomy; access to safe spaces; peer/family support; sensory environment; digital access; previous activity history	Preference-based modality; low logistical burden; environmental adaptation; behavior-change support; caregiver or peer support	Telehealth-supported activity; gamified mobile intervention; peer-assisted exercise; school/home/community programs; caregiver-mediated routines	MVPA; step count; sedentary time; attendance; retention; acceptability; maintenance after intervention	Focus on sustained participation rather than short-term performance; monitor whether digital or gamified tools increase anxiety or routine disruption	(4, 18, 26, 59, 60, 77, 78, 82, 91)
Safety, tolerability, and adverse-response prevention	Epilepsy or medical comorbidity where relevant; injury risk; fatigue; sensory overload; behavioral escalation; sleep worsening; caregiver burden	Conservative progression; safety monitoring; low initial sensory/social load; structured transitions; individualized adaptation	Any modality, but especially high-intensity, aquatic, competitive, or group-based programs should include explicit safety procedures	Adverse events; injuries; fatigue; sleep worsening; distress; behavioral escalation; dropout reasons; caregiver/instructor logs	Safety reporting should include both physical and behavioral adverse responses; reduce intensity, complexity, sensory load, or social demand when intolerance appears	(18, 23, 26, 51, 54, 77)

This decision model is research-informed rather than a validated clinical prescription algorithm. It is intended to help clinicians and researchers match exercise dose architecture to functional targets while explicitly monitoring feasibility, tolerance, and safety.

ASD, autism spectrum disorder; BOT-2, Bruininks-Oseretsky Test of Motor Proficiency, Second Edition; BRIEF, Behavior Rating Inventory of Executive Function; MABC-2, Movement Assessment Battery for Children, Second Edition; MVPA, moderate-to-vigorous physical activity; RPE, rating of perceived exertion; TGMD, Test of Gross Motor Development.

#### 3.4.1 Matching exercise dose to symptom domains and functional priorities

In order to translate clinical practices in ASD effectively, it’s important to let go of the belief that one exercise plan can achieve all therapeutic objectives equally. Current research reveals that exercise influences specific areas instead of having a universal effect, with more pronounced and consistent results for certain outcomes. Meta-analytic and systematic-review evidence indicates that physical activity interventions can improve motor competence, executive functions, sleep, and selected social-communication outcomes, but the magnitude and consistency of benefit vary by domain, intervention structure, and participant characteristics (4, 8, 9, 39). From a practical standpoint, exercise strategies for ASD should prioritize clinical targets like social engagement, behavior regulation, sleep enhancement, or motor skill development over the exercise type.

##### 3.4.1.1 Domain-guided prescription instead of modality-guided prescription

When the goal is to improve social communication and engagement, activities that focus on interpersonal interactions and structured rules may be more advantageous than those done alone. Structured physical activity programs have improved social interaction and communication in children with ASD (33), while network meta-analytic evidence suggests that some socially embedded exercise formats may confer comparative advantages for sociability and communication outcomes (37). Similarly, mini-basketball training has been associated with gains in executive functions, core symptoms, social communication, and even white matter adaptation, implying that programs combining motor challenge, shared attention, and coordinated action may be especially relevant when the functional target extends beyond common fitness (15, 37).

Programs that are coordinative and mentally stimulating might be more suitable than those centered only on aerobic intensity when seeking to enhance executive control, inhibitory regulation, or cognitive flexibility. The executive-function meta-analysis by Liang et al. (7), the network meta-analysis by Hou et al. (36), the recent balance/executive-function synthesis by Li et al. (73), and the aquatic-exercise study showing concomitant gains in executive function and BDNF all support this logic (8, 36, 74). When it comes to stabilizing sleep, elements such as consistency, timing, and long-term patterns may be more important than social complexity, as suggested by studies and meta-analyses on exercise and sleep in autistic children (9, 51, 53). Similarly, Aquatic therapy, leisure programs, and prolonged exercise interventions may hold more clinical significance than symptom-focused treatments when prioritizing quality of life and engagement (75, 83, 84). Overall, the goal of translation is not to find a single ‘best’ exercise, but to align dose structure with clinical objectives.

#### 3.4.2 Selecting clinically meaningful outcomes beyond global symptom scores

A further difficulty in translating research is that numerous ASD exercise studies continue to overly depend on overall symptom scores or common autism totals as their main outcomes. While those measures are still useful, they are often too broad to detect the most clinically important impacts of exercise. Current evidence suggests that exercise may exert its most reliable benefits on proximal functional systems—such as executive control, sleep quality, motor competence, social participation, arousal regulation, and family-perceived quality of life—rather than uniformly reducing all autism-related symptoms to the same degree (4, 8, 9, 36, 75). In a translational exercise trial, outcomes should be chosen according to the proposed mechanism and expected clinical benefits, instead of merely following tradition.

##### 3.4.2.1 From broad autism severity to proximal functional endpoints

For instance, when the intervention focuses on boosting cognitive control and neural plasticity, outcomes linked to executive function and corresponding biological or neuroimaging markers might reveal more than just the aggregate symptom scores. The aquatic-exercise BDNF study, the executive-function meta-analysis, and the mini-basketball/white-matter research collectively demonstrate that improvements in behavior can be linked with observable biological or neural-system changes (8, 15, 36). For interventions aimed at improving sleep and self-regulation, metrics such as sleep efficiency, wake-after-sleep-onset, sleep quality, and melatonin-related indices hold more clinical significance than standard autism questionnaires (9, 51, 53). To improve motor participation and daily functioning, center should be on balance, basic movement skills, objectively analyzed physical activity, and fitness linked to health (74, 85).

##### 3.4.2.2 Participation, quality of life, and family-level outcomes

Equally significant, translating clinical findings in ASD must center on outcomes that are important to families and influence daily life. Aquatic therapy has been associated with improvements in social competence and quality of life (79), a leisure program improved quality of life and stress in individuals with ASD (80), and a longer-term exercise intervention has been linked to improvements in metabolic profile, autism traits, and parent-perceived quality of life (74). Studies on family-focused physical activities emphasize outcomes such as objectively measured activity, movement skills, parental confidence, parental support, and shared activities as key translational endpoints, especially for younger children (4). These studies together suggest that future research should center on whether exercise enhances sleep, self-regulation, participation, family burden, and developmental opportunities, instead of just altering symptom scores.

#### 3.4.3 Adherence, safety, sensory adaptation, and family-level feasibility

Translation is unsuccessful if the set program cannot be maintained. This is especially crucial in ASD, as adherence is influenced by much more than just motivation. Recent reviews show that physical activity participation is influenced by barriers and facilitators across the individual, family, environmental, and social levels, including sensory sensitivities, limited adapted opportunities, social discomfort, low confidence, transportation issues, caregiver burden, and restricted access to autism-informed instructors (7, 22, 61). A review on behavior change indicates that this field has not consistently integrated techniques necessary for sustaining long-term physical activity in autistic individuals (26). Thus, rather than being seen as a secondary implementation issue, adherence should be regarded as a primary factor since it plays a vital role in the effective dose received.

##### 3.4.3.1 Sensory adaptation and environmental fit

Sensory adaptation exemplifies why typical exercise translation needs adjustment for ASD. In ASD, variations in sensory processing are commonly seen, and analyses of broader ASD treatments show that focusing on sensory issues can affect behavioral control, tolerance, and involvement (40, 41). This has direct implications for exercise design: aquatic programs may be beneficial for some children because buoyancy, pressure, and environmental predictability create a more tolerable sensory context, whereas noisy, chaotic, or rapidly changing group environments may increase withdrawal or dysregulation in others (79). Consequently, exercise recommendations ought to routinely encompass intensity, duration, sensory cues, environmental stability, instructional aid, and organized transitions.

##### 3.4.3.2 Family feasibility and delivery models

The practicality at the family level is just as important, particularly during childhood. Programs that include parents and families show that caregivers can stay involved and develop skills when there are clear support systems and reasonable expectations. Randomized or feasibility work on parent-mediated fundamental motor skill interventions, preschool family-based physical activity programs, and caregiver-supported delivery models all suggest that home- and family-linked formats can improve uptake and practicality (79, 80). Telehealth has further broadened this area of translation: the PLANE program for children and caregivers, telehealth-delivered peer-assisted physical activity for autistic adults, and parent-perspective studies on mediating physical activity all indicate that remote or hybrid models can reduce access barriers while preserving acceptability (4, 62). The main lesson in clinical practice is that the most beneficial exercise plan isn’t necessarily the one with the highest potential gains, but the one that can be safely carried out, comfortably maintained, and consistently woven into family schedules.

#### 3.4.4 Translating exercise programs across childhood, adolescence, and adulthood

The use of exercise in the development of ASD shows varying results. Most intervention studies concentrate on childhood, where the evidence is fairly strong, and these programs frequently include parental involvement, school partnerships, or skill enhancement (4, 79, 80). This makes sense developmentally because early and middle childhood offer great opportunities for acquiring motor skills, forming routines, and engaging with the help of caregivers. Simultaneously, this field commonly assumes that evidence from childhood applies widely throughout life, despite important changes in exercise barriers, motivations, and outcomes during adolescence and adulthood.

##### 3.4.4.1 Childhood and adolescence

During childhood, the most practical models for translation seem to be those that incorporate exercise into family, school, or organized skill-learning environments. Programs that include physical activity and motor skills, led by parents and aimed at preschool and school-age children, have shown to be feasible and initially effective (79, 80). However, during adolescence, translation becomes more complex. A recent scoping review on motivation to participate in structured physical activity emphasizes that autistic youth face distinct motivational and contextual barriers, while longitudinal sport-participation data suggest that autistic children and adolescents are more likely to follow low-participation trajectories than their non-autistic peers (86, 87). This suggests that exercise programs for teenagers should emphasize autonomy, social integration, identity, and perceived competence more than those for children.

##### 3.4.4.2 Adulthood and the underdeveloped translational pipeline

The disparity is most apparent in adulthood. The systematic review by Pan et al. makes clear that young adults with ASD remain an underserved population, with the strongest evidence limited to physical fitness, motor outcomes, psychological function, and quality of life, and much weaker evidence for changes in physical activity levels or core autism symptoms (81). Complementary work on adult participation shows persistent barriers related to access, comfort, and context (59), while telehealth-based peer-assisted physical activity studies suggest feasible delivery models but still represent early-stage translation instead of mature implementation (60). Creating distinct protocols for both youths and adults is a significant step forward, but it highlights the slow progress in expanding age-specific treatments (21). A precision model that considers the entire lifespan will need different entry points at various developmental stages: movement promoted by family during childhood, structured activities tailored to motivation in adolescence, and models promoting autonomy and access for real-world participation in adulthood.

#### 3.4.5 Integrating exercise into multimodal ASD care pathways

Exercise should not be treated as an isolated component separate from the overall care for ASD. A practical clinical method is to include physical activity in a comprehensive plan that also covers sleep management, caregiver education, nutrition and weight management, anxiety therapy, and developmental-behavioral support. Integrative care reviews already argue for multimodal management in autism instead of single-intervention thinking, and the sleep literature in ASD likewise shows that behavioral approaches, sleep hygiene, melatonin, and related rhythm-focused strategies are often combined instead of used in isolation (85). Within this framework, exercise should be considered as a part of a larger therapeutic system.

##### 3.4.5.1 Exercise plus sleep-focused care

Sleep is one of the most obvious opportunities for integration. Organized physical activities, according to research and exercise trials, can enhance sleep quality in children with autism, potentially providing benefits similar to those of melatonin in certain situations (9, 51, 53). instead of setting exercise against pharmacological or behavioral sleep treatments, the more useful clinical question is how exercise timing, frequency, and adherence can be combined with bedtime routines, sensory accommodations, or melatonin when appropriate. Exercise could be a likely upstream regulator in sleep-focused care strategies, particularly for children experiencing disruptions in both daytime alertness and nighttime sleep.

##### 3.4.5.2 Exercise plus caregiver-mediated and digital care

Caregivers offering digital support is another integration approach. Telehealth has quickly expanded to train caregivers of autistic children (86), and systematic reviews show that well-designed telehealth can effectively aid a range of child-focused ASD interventions (87). In the realm of exercise-related care, the PLANE initiative merges physical activity with nutritional education for both children and their caregivers, and a parent-focused behavioral intervention has been tailored for children with ASD and overweight (88). By embedding physical activity into daily life, parenting strategies, and health behavior adjustments, these models play a vital role, instead of viewing it as an isolated ‘sports matter.

##### 3.4.5.3 Exercise plus mental-health and behavior-focused interventions

A third opportunity lies at the interface with anxiety and self-regulation treatment. Telehealth-delivered CBT and family-based internet-delivered CBT programs have shown feasibility in autistic youth with anxiety and/or OCD (89), revealing that future care models could strategically combine structured physical activity with CBT-based anxiety management, especially in adolescents for whom anxiety, avoidance, or social overload limits participation. Seen from this angle, exercise is not merely an extra activity but a modifiable behavioral core that can be incorporated with sleep management, caregiver advice, nutritional support, and mental health counseling. Thus, the aim of translation should be to integrate instead of isolate: exercise plans for ASD are likely to be most successful when integrated into coordinated, phenotype-aware, and family-friendly multimodal care strategies.

#### 3.4.6 Safety, adverse events, and risk monitoring

Safety should be treated as a core component of ASD exercise prescription rather than as a secondary implementation detail (12, 13, 22, 23, 44). Although many exercise interventions in ASD appear feasible, the literature has not consistently reported adverse events, distress, sensory overload, behavioral dysregulation, injury, fatigue, or dropout reasons (22, 23, 27, 42, 43, 52, 79). This limits the ability to estimate risk across exercise modalities and dose architectures (22, 23, 27, 44). Future trials should systematically record musculoskeletal injuries, excessive fatigue, cardiovascular symptoms, behavioral escalation, sensory intolerance, sleep worsening, refusal, dropout, and caregiver burden (22, 23, 44, 51, 52, 79). Risk monitoring is especially important when programs involve high sensory load, rapid transitions, competitive group settings, high-intensity exercise, or participants with epilepsy, motor impairment, anxiety, sleep disturbance, obesity, or other medical comorbidities (5, 9, 13, 22, 30, 40, 41, 61, 68).

### 3.5 Future directions for precision exercise science in ASD

#### 3.5.1 Standardizing dose reporting and intervention taxonomy

One important barrier to ongoing advancements in ASD exercise science is the continuous absence of standardized dose reporting. Even though recent reviews now provide stronger guidance on exercise prescription in autism, this field still reports interventions using inconsistent combinations of modality labels, session duration, frequency, or broad descriptors such as “moderate intensity”, without adequately characterizing progression, adherence, sensory context, social demand, cognitive load, or actual delivered exposure (2, 12). Consequently, interventions that differ significantly in biological and behavioral aspects are often categorized under the same label, complicating cross-trial comparisons and weakening meta-analytic conclusions. In the future, a taxonomy should look beyond just the mode and see exercise as a multifaceted exposure, marked by physiological load, motor complexity, social integration, sensory structure, temporal patterns, and implementation challenges.

Future studies should at least document the planned dose, the administered dose, the tolerated dose, and the dose sustained over time. For ASD in particular, the framework should incorporate intervention architecture: Whether the session is for one person or a group, follows a consistent pattern or changes, is tailored for sensory needs or not, and includes altering rules, imitation, collaboration, or sustaining a common focus. Such a taxonomy would make it possible to compare programs that are superficially different but mechanistically similar, while also distinguishing programs that share a modality label yet impose very different demands. Standardized reporting is therefore not a technical afterthought; it is the prerequisite for building a credible precision-exercise literature in ASD (2, 12).

#### 3.5.2 Designing biomarker-informed and mechanism-linked trials

To provide clearer guidance for investigators, we prioritize future research directions according to likely clinical impact and feasibility. **Table 5** outlines Prioritized research agenda for precision-informed ASD exercise science.

**Table 5:** Prioritized research agenda for precision-informed ASD exercise science.

Priority	Research direction	Specific actions needed	Clinical impact	Feasibility	Rationale	Literature support
1	Standardize exercise dose reporting	Report frequency, intensity, time, type, physiological load, motor complexity, cognitive demand, social embeddedness, sensory load, predictability, progression rate, environmental adaptation, adherence, and delivered/tolerated/sustained dose	High	High	Inconsistent dose reporting is one of the main barriers to comparing ASD exercise studies and developing individualized prescriptions	(9, 11, 18, 26, 52, 53)
2	Systematically report safety, adverse events, and non-response	Record injuries, fatigue, sleep worsening, sensory overload, behavioral escalation, refusal, dropout reasons, caregiver burden, and other adverse responses	High	High	Safety and tolerability are essential for clinical translation, but adverse responses and dropout reasons are inconsistently documented	(18, 23, 26, 51, 54, 77)
3	Include null, inconsistent, and adverse findings in evidence synthesis	Report non-significant outcomes, acute exercise findings, domain-specific inconsistency, and potential transient worsening	High	High	A balanced evidence base prevents overstatement and clarifies which outcomes are most responsive to exercise	(3, 5–8, 10, 20, 23)
4	Improve sex-aware recruitment, reporting, and analysis	Report male/female numbers and percentages; recruit sufficient girls and women; conduct planned sex-stratified or sex-aware analyses when sample size permits	High	Moderate	Female underrepresentation limits generalizability and prevents firm conclusions about sex-related exercise response	(1, 2, 6, 14, 57, 58, 61)
5	Stratify studies by developmental stage and pubertal status	Distinguish childhood, adolescence, and adulthood; document pubertal stage, age at menarche where relevant, sleep changes, fatigue, body awareness, and internalizing symptoms	High	Moderate	Chronological age alone is insufficient because puberty may alter sleep, mood, social experience, sensory tolerance, and exercise adherence	(6, 14, 16, 65–67, 87, 88)
6	Operationalize exercise dose architecture	Use structured coding for physiological load, motor complexity, cognitive demand, social embeddedness, sensory load, predictability, progression, environmental adaptation, and adherence burden	High	Moderate	The proposed precision framework depends on making dose architecture measurable rather than descriptive	(9, 11, 18, 26, 52, 53)
7	Design mechanism-linked ASD exercise trials	Combine behavioral outcomes with feasible mechanistic readouts such as BDNF, actigraphy, sleep measures, inflammatory markers, heart-rate-derived indices, and neuroimaging in selected subsamples	High	Moderate	Current mechanistic claims remain partly indirect; future trials should test whether specific exercise architectures engage specific biological or behavioral pathways	(12, 33, 35, 48–51, 90)
8	Match outcome measures to target domains	Select proximal outcomes according to the intended mechanism and clinical target, such as sleep efficiency for sleep interventions, executive-function tasks for cognitive interventions, or participation measures for social and quality-of-life interventions	High	High	Global autism symptom scores may miss clinically meaningful changes in sleep, motor competence, executive function, participation, family burden, or quality of life	(3, 7, 20, 33, 73, 74, 79–81)
9	Conduct dismantling and comparative dose-component studies	Compare interventions that differ in one major dimension, such as social embeddedness, motor complexity, sensory load, or predictability, while holding other components relatively constant	Moderate to high	Moderate	Current studies often compare modalities rather than active ingredients, making it unclear whether benefits arise from aerobic load, motor learning, social interaction, or environmental fit	(9, 11, 34–37, 43, 48)
10	Expand adult and lifespan-focused exercise research	Develop age-appropriate protocols for adolescents, young adults, and older autistic adults; examine autonomy, community access, health-related fitness, mental health, and long-term participation	High	Moderate	The evidence base remains child-centered, while adults face distinct barriers to physical activity participation and service access	(6, 59, 60, 78, 81, 83, 84)
11	Strengthen implementation science and real-world scalability	Measure reach, adoption, fidelity, instructor training, cost, caregiver burden, retention, sustainability, and maintenance after intervention ends	High	Moderate	An intervention with short-term efficacy has limited value if it cannot be sustained in homes, schools, clinics, or community settings	(4, 18, 19, 26, 59, 60, 77, 78, 82, 91, 92)
12	Develop caregiver-mediated, school-based, community-based, and telehealth-supported models	Test flexible delivery formats that reduce transportation, cost, instructor-access, and scheduling barriers while preserving core intervention components	High	Moderate	Family and environmental constraints strongly shape whether the prescribed dose becomes a delivered and sustained dose	(4, 18, 26, 60, 77, 82, 86, 87, 91)
13	Integrate exercise with multimodal ASD care pathways	Study exercise as part of combined care involving sleep management, caregiver education, nutrition, behavioral support, anxiety treatment, and digital health tools	Moderate to high	Moderate	Exercise is unlikely to function optimally as an isolated intervention when sleep, anxiety, sensory processing, family context, and health behaviors interact	(50, 85–89, 91)
14	Test gut-brain and microbiome mechanisms in human ASD studies	Combine exercise dose documentation with diet monitoring, gastrointestinal symptoms, microbiome profiling, metabolomics, and behavioral outcomes	Moderate	Low to moderate	Animal-model evidence is promising, but human ASD exercise-microbiome evidence remains insufficient for clinical interpretation	(13, 54, 55)
15	Build larger, preregistered, adequately powered trials	Use predefined primary outcomes, transparent allocation, longer follow-up, planned subgroup analyses, and standardized reporting of intervention fidelity and harms	High	Moderate to low	Many existing studies are limited by small samples, short duration, heterogeneous outcomes, and weak reporting of dose, fidelity, and adverse events	(3, 5–10, 20)

**Note:** Clinical impact reflects the likely importance of the research direction for improving ASD exercise prescription, safety, individualization, or implementation. Feasibility reflects the practical likelihood that the research direction can be implemented in near-term studies using currently available tools and designs. Priorities are intended to guide future research planning rather than to establish clinical guidelines.

**Abbreviations:** ASD, autism spectrum disorder; BDNF, brain-derived neurotrophic factor.

The future direction of this field should move from focusing solely on outcome efficacy to trials that are connected to mechanisms. Current ASD exercise research already points to several plausible biological and systems-level pathways, including trophic signaling, white-matter adaptation, sleep-arousal regulation, inflammatory modulation, and gut-brain signaling, but most studies still measure behavioral change without testing whether the hypothesized mechanism has actually been engaged (2, 14, 15, 36). This gap is important because exercise is unlikely to yield a uniform therapeutic outcome. Various exercise structures might activate distinct pathways, and without designs informed by biomarkers, this diversity remains hidden.

Trials related to mechanisms in ASD don’t have to start with highly invasive biomarker panels. A pragmatic first step would be to combine behavioral endpoints with proximal mechanistic readouts that are feasible and scalable, such as actigraphy, sleep metrics, heart-rate-derived indices, blood-based trophic or inflammatory markers, and targeted neuroimaging in a subset of participants (14, 15, 36, 90). Research indicating exercise-induced alterations in BDNF, white matter integrity, and the practicality of actigraphy implies that these designs are already feasible in ASD groups (15, 36, 90). The methodological priority is to test explicit mediation pathways—for example, whether a given dose improves executive function through measurable changes in sleep regularity, neurotrophic signaling, or network-level adaptation—rather than simply asking whether pre-post scores improve.

#### 3.5.3 Building sex-stratified and developmental-stage-stratified studies

A precision method to ASD will be lacking unless research is intentionally divided by sex and developmental stage. The existing body of evidence remains predominantly centered on males, although existing developmental study suggests that initial sex differences in phenotype might be less pronounced than previously thought, with more noticeable differences emerging later in development (17, 18). Exercise science is directly influenced by this: a study that fails to consider puberty status, gender-related phenotype expression, and age-related participation requirements is unlikely to uncover key subgroup differences, even if they are present. Future research should no longer consider sex and age as mere background factors but rather as key design elements.

In practical terms, this means recruiting enough girls and women to support planned analyses, characterizing pubertal stage instead of age alone in adolescent samples, and distinguishing childhood, adolescence, and adulthood as physiologically and behaviorally distinct phases of exercise responsiveness (6, 17, 18). Stratification according to developmental stages is crucial since puberty affects sleep, mood, social awareness, fatigue, and tolerance for participation, which can change how an exercise dose is perceived and maintained. this field should shift towards trial templates that consider sex and stage, instead of relying on pooled samples that lack the power to address heterogeneity.

#### 3.5.4 Advancing implementation science and real-world scalability

Even the most well-constructed efficacy studies will have limited clinical significance if they cannot be applied in real-world environments. In the case of ASD, it is particularly noticeable that physical activity participation is impacted by a range of barriers at the individual, family, provider, environmental, and service system levels (6, 22, 23, 62). Current evaluations emphasize hurdles including a deficiency in personalized opportunities, sensory variations, transportation and scheduling difficulties, lack of provider readiness, and uneven application of behavior-change strategies. Simultaneously, broader autism implementation science suggests that the gap between research and practice is a key issue across various intervention areas, not just in exercise (22–24, 62, 81, 92).

In ASD exercise science, implementation should be treated as a main outcome rather than an afterthought. Future studies should consistently document adoption, reach, training demands, expenses, retention, negative incidents, family workload, and sustainability following the conclusion of formal intervention. Models supported by telehealth and assisted by technology could be particularly beneficial in this context. Preliminary studies on adults with autism and child interventions supported by families suggest that telehealth, parental assistance, and gamified mobile platforms can improve feasibility and accessibility, though their long-term impact remains unverified (23, 62, 81, 82). The most effectively scalable interventions are probably those that integrate a structured, evidence-based strategy with flexible delivery methods, allowing them to be implemented in homes, schools, clinics, and communities while preserving their essential components.

#### 3.5.5 A precision framework for the next generation of ASD exercise research

Overall, this field is prepared to transition from a common efficacy model to a precision-exercise approach for ASD. In that framework, exercise is not treated as a uniform wellness activity but as a structured developmental exposure whose effects depend on dose architecture, mechanism engagement, developmental timing, sex-related heterogeneity, and real-world implementability (2, 6, 12, 14, 17, 18, 22–24, 62, 81, 92). The main scientific inquiry is no longer about whether exercise is effective for ASD in general. Aligning a specific exercise framework with a therapeutic aim for a particular subgroup involves using outcome measures that are both mechanistically comprehensible and clinically relevant.

**Figure 3:**
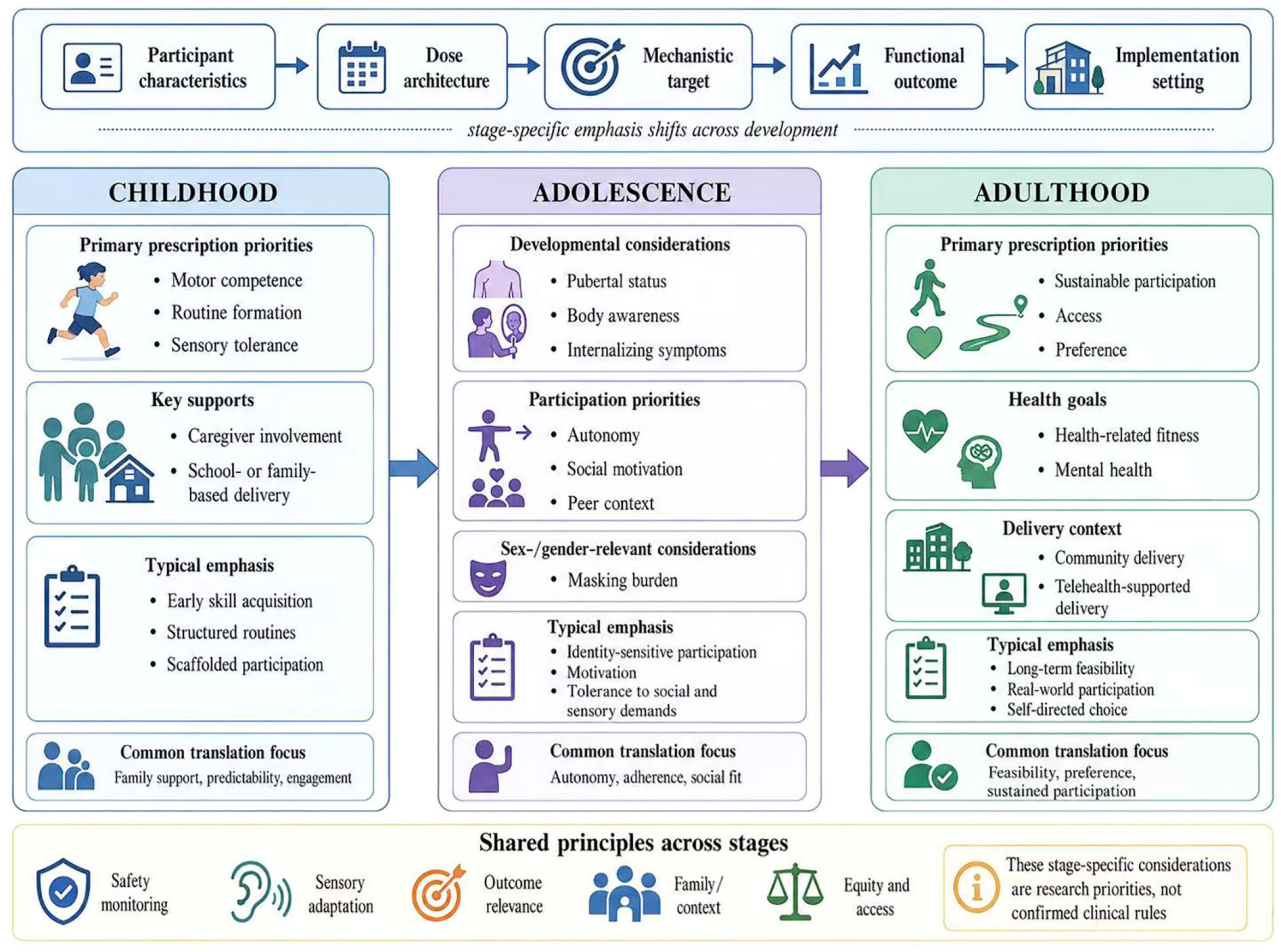
The model illustrates how exercise prescription priorities may shift across developmental stages. In childhood, emphasis may be placed on motor competence, routine formation, caregiver involvement, sensory tolerance, and school- or family-based delivery. In adolescence, pubertal status, autonomy, body awareness, internalizing symptoms, social motivation, masking burden, and peer context become increasingly important. In adulthood, prescription should emphasize sustainable participation, access, preference, health-related fitness, mental health, and community or telehealth-supported delivery. These stage-specific considerations are proposed as research priorities rather than confirmed clinical rules.

A useful precision model would connect five layers: participant characteristics, dosage structure, mechanistic target, functional outcome, and implementation setting. Age, puberty stage, sex-related trait expression, motor skills, sleep patterns, sensory tolerance, and family background should be part of the participant profile. Dose architecture should encompass not just intensity and frequency, but also motor complexity, social demands, predictability, and sensory load. In the trial-design phase, it is important to clearly define mechanistic targets and include functional endpoints beyond just overall symptom scores, such as participation, sleep, executive function, self-regulation, and quality of life. Ultimately, the context of implementation should decide if the intervention can be sustainably maintained. In this model, the future of ASD exercise science focuses on developing a stratified, mechanism-aware, and scalable intervention science instead of creating more varied short-term trials. **Figure 3** illustrates a precision exercise translation model for ASD throughout developmental stages.

### 3.6 Null, inconsistent, and potentially adverse findings

The evidence base should not be interpreted as uniformly positive (2, 4, 6, 8–12). Some outcomes show inconsistent effects, and acute exercise cannot be assumed to produce the same benefits as chronic training (6, 8, 9, 11, 27). For example, one randomized cross-over study found that a single 20-minute bout of moderate aerobic exercise did not improve affective response inhibition and transiently worsened aspects of socio-emotional processing in children with ASD (23). These findings support a dose-architecture perspective: exercise effects may depend on timing, intensity, social and sensory context, developmental stage, and the outcome being measured (12, 13, 22, 23, 27). They also justify the need to report adverse responses and non-response rather than only beneficial outcomes (22, 23, 27, 44).

## 4 Conclusion and outlook

Exercise for autistic individuals should not be reported merely as a generic lifestyle activity, but as a structured and measurable intervention exposure. The current evidence supports a more cautious interpretation: exercise may become a precision-informed developmental strategy if future studies specify dose architecture, test mechanism engagement, stratify developmental and sex-related heterogeneity, and evaluate real-world feasibility. Across the literature, exercise-based interventions have shown the potential to improve multiple ASD-relevant domains, including executive function, sleep, motor competence, repetitive behaviors, social communication, and broader aspects of participation and quality of life. At the same time, these benefits are clearly not uniform, and this field has now reached the point where the central question is no longer whether exercise is beneficial in a general sense, but which exercise dose works best, for whom, at what stage of development, and through which pathways. A major takeaway from this review is that the exercise dose in ASD should be considered a multifaceted concept, extending beyond just modality or the traditional FITT descriptors. Although physiological load is important, it represents only a portion of the overall significance. Motor complexity, cognitive demand, social embeddedness, sensory load, predictability, adherence burden, and contextual fit are also likely to shape whether an exercise program becomes physiologically active, behaviorally tolerable, and clinically effective. This rephrasing clarifies why interventions that appear similar can produce varying results, and why it has been challenging for this field to pinpoint a universally optimal program.

From a mechanistic perspective, existing research endorses a model with multiple pathways. Regular physical activity could impact functions related to ASD by enhancing neuroplasticity, changing synaptic structures, adjusting sleep and arousal, affecting neuroinflammation and immunometabolism, influencing gut-brain interactions, and enhancing cognitive-motor integration. However, the extent to which these pathways contribute likely varies among individuals and different intervention designs. Therefore, future research should progress from broad efficacy studies to trials that examine if particular exercise structures activate specific biological and behavioral targets. Without these designs, mechanistic heterogeneity will be assumed rather than proven. This review highlights the importance of considering both sex and developmental stage to understand variability in exercise response. The available evidence is restricted by the insufficient inclusion of girls, women, and adults, and the usual method of grouping participants from diverse developmental stages. Recent findings indicate that differences in sex-related ASD differences might be influenced by factors beyond biology, including the timing of diagnosis, social masking, puberty, internalizing challenges, and developmental context. To successfully implement precision exercise science in ASD, studies need to integrate sex-specific insights, developmental stratification, and emphasize outcomes that are relevant to various autism phenotypes. From a practical standpoint, the future of this field depends on connecting effectiveness with practicality. Exercise strategies need to align with symptom areas and functional targets, be measured by clinically relevant outcomes, and account for adherence, sensory adaptation, family context, and service-delivery constraints. Interventions that are not sustainable in daily life will have limited clinical significance, even if they are effective in the short term in controlled studies. Consequently, the research agenda ought to focus on implementation science, caregiver-mediated models, telehealth services, and the incorporation of sleep, mental health, and behavioral care. At present, the framework should be interpreted as hypothesis-generating rather than as a finalized clinical prescription model. Its value lies in clarifying what future ASD exercise trials should measure, report, stratify, and monitor.

## Data Availability

The original contributions presented in the study are included in the article/supplementary material. Further inquiries can be directed to the corresponding author.
